# Evaluating the immunogenicity of a mouse partial hindlimb for composite allotransplantation

**DOI:** 10.3389/fimmu.2025.1595319

**Published:** 2025-06-18

**Authors:** Jia Hui (Angela) Sun, Samantha Ligi, Felor Biniazan, Siba Haykal

**Affiliations:** ^1^ Latner Thoracic Surgery Laboratories, University Health Network, Toronto General Hospital, Toronto, ON, Canada; ^2^ Institute of Medical Science, Temerty Faculty of Medicine, University of Toronto, ON, Canada; ^3^ Department of Surgery, Division of Plastic and Reconstructive Surgery, Yale School of Medicine and Yale New Haven Health, New Haven, CT, United States

**Keywords:** vascularized composite allotransplantation, immunogenicity, graft rejection, tolerance induction, tissue engineering

## Abstract

**Background:**

Vascularized composite allotransplantation (VCA) offers a promising solution for restoring function in patients with severe tissue trauma, yet acute rejection remains a major hurdle. Acute rejection is driven by an immune response against transplanted tissues, requiring lifelong immunosuppression, increasing risks of infections, malignancies, and organ toxicity. This study aims to develop a mouse hindlimb transplant model to investigate immune responses and the phenotype of infiltrating cells in these grafts.

**Materials and methods:**

Syngeneic and allogeneic mouse partial hindlimb models were established to evaluate grafted skin and muscle immunogenicity. Male BABL/c and C57BL/6 mice served as donors, C57BL6.sjl mice were recipients. Hindlimbs were procured, including femur, muscle, and skin, and were heterotopically transplanted into recipient mice. Samples were collected at post-operative days 7 (POD7) and 14 (POD14) for histological analysis (H&E staining, immunohistochemistry for CD8, caspase-3, CD31, and TUNEL) to assess cell infiltration, inflammatory T cell presence, apoptosis, and vascularization. Immune cell populations were characterized through flow cytometry.

**Results:**

Both syngeneic and allogeneic skin and muscle showed increased cellular content at both time points. Allogeneic skin at POD14 exhibited higher cellular content and subclinical rejection, while flow cytometry revealed increased donor-derived and recipient-derived T cells, particularly CD4+ and CD8+ T cells. Additionally, Tr1 cells were more abundant, suggesting a regulatory role in the immune response. Apoptotic markers increased in both grafts at POD14, with more TUNEL-positive cells in allogeneic grafts at POD7. Revascularization, assessed by CD31 expression, was notably present in both syngeneic and allogeneic muscle at POD14.

**Discussion:**

This study provides valuable insights into acute rejection in VCA using a novel mouse hindlimb transplant model. Findings reveal immune response complexity, with increased CD8+ T cells and Tr1 cells in allogeneic skin, and unexpected vascularization in non-vascularized grafts. The rise in Tr1 cells suggests a potential mechanism for immune regulation, offering potential for tolerance induction strategies. These results emphasize the need for tissue-specific immunosuppressive approaches, where targeting Tr1 cells could reduce dependence on broad immunosuppression, improving long-term graft survival and patient outcomes. This work lays the foundation for refining VCA therapies, with more personalized, less toxic immunosuppressive strategies.

## Introduction

Vascularized composite allotransplantation (VCA) is a life-enhancing procedure that involves the transplantation of multiple tissues, such as skin, bone, muscle, fat, nerves, and lymph nodes, to restore function and aesthetics in patients with severe tissue trauma or functional deficits. While VCA has been successfully applied to structures like limbs, faces, and reproductive organs, acute rejection remains a significant barrier, occurring in over 89% of recipients (1). Acute rejection is driven by a robust immune response against the transplanted tissue, necessitating lifelong immunosuppression with drugs like tacrolimus, steroids, and mycophenolate mofetil (MMF) (2). However, these regimens are associated with severe side effects, including infections, malignancies, organ toxicity, and reinnervation challenges, which can compromise graft functionality (3–5). Current immunosuppressive protocols, adapted from solid organ transplantation, fail to address the unique immunological complexity of VCA grafts, which consist of multiple tissue types with varying immunogenic properties (4). This highlights the urgent need for research into tolerance induction and less intensive immunosuppressive strategies to improve graft outcomes.

The skin, being highly immunogenic, is a primary target of rejection in VCA, driven by immune cells such as Langerhans cells, γδ T cells, and tissue-resident memory T cells (TRM) (6–11). Vascular components, while critical for graft viability, are also vulnerable to immune-mediated damage, leading to conditions like graft vasculopathy (12–14). Other tissues, such as bone, muscle, and nerves, exhibit varying degrees of immunogenicity and rejection patterns, contributing to the complexity of VCA immunology (15–18). Understanding these tissue-specific immune interactions is essential for developing targeted therapies to improve graft survival.

To address these challenges, this study developed a mouse heterotopic partial hindlimb transplant model to study these immune responses. The use of a mouse model allows for the characterization of immune cell populations within the graft and provides a platform to investigate tissue-specific immune interactions using a custom 16-color flow cytometry panel (**Supplementary Figure 1**). We have implemented congenic strains for donor and recipient mice to track cell origin. The donor BALB/c and C57BL/6 strains will express CD45.2 in all hematopoietic cells, and the recipient C57BL/6.SJL strain will express CD45.1 in all hematopoietic cells. Additionally, our flow cytometry panel allows for characterization of various immune cell populations such as NK cells, B cells, CD4+ and CD8+ T cells, as well as cytotoxic and regulatory T cell subpopulations. Histological analysis using hematoxylin and eosin (H&E) staining, as well as TUNEL and immunohistochemistry staining for CD8, caspase-3, and CD31 will reveal immune cell localization within the graft cellular apoptosis, and tissue revascularization. These findings will provide critical insights into the immune mechanisms driving acute rejection and identifying cells as a potential therapeutic target for tolerance induction in VCA.

By elucidating the mechanisms underlying acute rejection and identifying key immune modulators, this work aims to pave the way for more effective and less toxic immunosuppressive strategies in VCA.

## Materials and methods

### Animal use

Male BABL/c, C57BL/6, and C57BL6.sjl mice 4 to 6 weeks old and weighing 20g to 25g were obtained from The Jackson Laboratory. All procedures were performed in compliance with guidelines by Animal Care Committee at Toronto General Hospital Research Institute, University Health Network (Toronto, ON, Canada). BABL/c and C57BL/6 both express CD45.2 in all hematopoietic cells and were used as donors, while C57BL6.sjl express CD45.1 in all hematopoietic cells and were used as recipients. This allows for the differentiation between donor and recipient cells identified within grafts for the purpose of tracking cell origin. A total of 7 BABL/c and 7 C57BL/6 mice were used as donors and 28 C57BL/6.SJL mice were used as recipients, resulting in 28 total transplantations performed. Both hindlimbs were utilized from the donor mouse.

### Mouse hindlimb procurement

For all surgeries, sterile and standard microsurgical techniques were employed. The surgical technique for donor (BALB/c, C57BL/6) hindlimb procurement is as follows: After removing hair from both hindlimbs and preparing the area with Betadine surgical scrub and 70% ethanol, an incision was made at the inguinal ligament level using a #10 scalpel blade, moving from lateral to medial direction. Once the underlying fat and muscle layers were exposed, blunt dissection was used to carefully dissect around the femur. A bone cutter was then used to transect the femoral bone. After isolating the hindlimb, the bone cutter was used again to separate the upper and lower hindlimb at the knee. Excess skin and fat were trimmed from the upper hindlimb using dissecting scissors before transplantation. The remaining hindlimb consisted of the whole femur, surrounding muscle, and a patch of skin approximately 2 cm in diameter.

### Heterotopic transplantation

Recipient mice (C57BL/6.sjl) were anesthetized using 5% isoflurane in oxygen at 1 L/min for induction and maintained at 2% in oxygen at 1 L/min. The mouse was placed on a water recirculating warming pad to maintain body temperature throughout the procedure. After removing hair from both hindlimbs and preparing the area with Betadine surgical scrub and 70% ethanol, a 0.5–1 cm incision was made on the upper dorsal region of the recipient mouse, along the cervical spine. Scissors were used to detach the skin from the underlying muscle layers, and the graft was implanted subcutaneously with the skin exposed. The skin was sutured to the recipient’s skin with interrupted sutures using #6–0 non-absorbable surgical sutures. Tegaderm, a transparent dressing, was trimmed into thin strips and wrapped around the entire thoracic region of the mouse. Following the procedure, the recipient mouse was returned to a clean, warm, and dry cage with water provided for the following 3 days, along with regular food. Tacrolimus was administered daily at a dose of 5 mg/kg intraperitoneally for both syngeneic and allogeneic recipient mice until the end of the monitoring period. Mice were sacrificed at 7- and 14-days post-operative, and the grafts were then procured and separated into skin, muscle, and femur for histological and flow cytometry analysis.

### Histology and immunohistochemistry

Native and grafted skin, muscle, and femur biopsies were taken at the time of graft procurement and fixed in 10% neutral buffered formalin and transferred to 70% ethanol before embedding in paraffin. The paraffin sections were cut at 5 μm using a microtome (Leica Biosystems) for hematoxylin and eosin (H&E) and immunohistochemical staining. Specifically, the paraffin samples were cleared and rehydrated through a series of xylene and ethanol and staining with hematoxylin (Sigma-Aldrich, HHS32) and eosin (VWR, 71304) to visualize the graft morphology and cellular infiltration.

Presence of CD31, CD8, and caspase-3 was evaluated in skin and muscle using CD31 recombinant rabbit monoclonal antibody (1:100, ThermoFisher, MA5-38125), CD8 recombinant rabbit polyclonal antibody (1:200, ThermoFisher, PA5-88265) and caspase-3 recombinant rabbit monoclonal antibody (1:50, ThermoFisher. 700182). Positive controls included native mouse lung, native mouse lymph node, and native mouse colon for CD31, CD8, and caspase-3, respectively (**Supplementary Figure 2**). Antigen retrieval was performed via heat induction in 1x citrate buffer (Abcam, ab93678), followed by permeabilization with 0.2% Triton X-100 in PBS at room temperature (20-min) and a PBS wash. Blocking was performed with 5% BSA solution (ThermoFisher, 15260-037) for 30-min and the primary antibody was incubated overnight at 4˚C. A 10-min incubation in 3% H_2_O_2_ (v/v) (Sigma-Aldrich, H1009) was performed to deactivate endogenous peroxidase followed by a 10-min PBS wash. Slides were then incubated with HRP anti-rabbit IgG secondary antibody (1:500, Abcam, ab288151) for 1h followed by another 10-min PBS wash. Then, 30 μL of DAB chromogen was diluted in 1.5 mL DAB substrate (Abcam, ab64238) and applied to the slides. Samples were counterstained with hematoxylin solution, washed in tap water, dehydrated in xylene, cleared, and mounted.

Whole stained H&E and immunohistochemical slides were scanned with the AperioScope CS2 slide scanner (Leica Biosystems) and viewed with Aperio Imagescope digital pathology software (Leica Biosystems). Allograft rejection was scored according to the Banff 2007 working classification of skin-containing composite tissue allograft pathology (19).

### TUNEL staining

To assess apoptosis in the 5 μm sections obtained from the skin and muscle, terminal dexoynucleotidyl transferase 2’-deoxyuridine 5’-triphosphate nick end labeling (TUNEL) assay was performed with the *In Situ* Cell Death Detection Kit, TMR red (Roche, 12156792910). The slides were incubated in a humidified chamber for 20-min at 37°C and covered in 10 μg/mL proteinase K (Abcam, ab64220) in 10mM Tris/HCl. The TUNEL reaction mixture was added to each slide and samples were incubated for 1h at 37°C, followed by a 5-min incubation with 10 μg/mL DAPI (Sigma-Aldrich, 10236276001) at room temperature. Samples were then mounted with ProLong Gold Antifade Mountant (ThermoFisher, P36930). Label solution without terminal transferase was used for the negative controls and positive controls were incubated in 1 mg/mL DNase I solution (50 mM Tris-HCl, pH 7.5, 1 mg/mL BSA) for 10-min at room temperature before the labeling procedure (**Supplementary Figure 3**). Stained slides were then scanned with the Widefield-Zeiss AxioObserver.

### Tissue processing

Immediately following procurement, syngeneic and allogeneic graft skin and muscle were stored in cold FACS buffer (2% FBS and 2mM EDTA in PBS) until dissociation into single cell suspension for flow cytometry. The skin was digested using DNAse I (Stemcell Technologies. 07900), collagenase MA (VitaCyte, 001-2030), and AOF BP protease (VitaCyte, 003-1000), in RPMI 1640 culture medium (ThermoFisher, 11875093). The skin tissue was cut into small pieces using scissors, incubated at 37°C for 10 minutes, cut into finer pieces, and again incubated at 37°C for 10 minutes. The muscle was digested using the Miltenyi Mouse Skeletal Dissociation Kit for mouse and rat (Miltenyi Biotec, 130-098-305), in a gentleMACS tissue dissociator according to manufacturer’s instructions (Miltenyi Biotec, 130-093-235).

### Flow cytometry analysis

Following the dissociation process, the cells were filtered through a 70 µm filter and centrifuged at 400 x g and 4°C for 10 minutes for the skin and 20 minutes for the muscle. Cells were then stained with Fixable Viability Stain 575v (BD Biosciences, 565694) at a 1:1000 dilution in PBS and incubated at 4°C, protected from light, for 10 minutes. This was followed by a 10-minute incubation in a blocking solution, after which the cells were washed and stained with a cocktail of fluorochrome-conjugated monoclonal antibodies targeting 13 extracellular markers (**Supplementary Table 1**). Subsequently, the cells were fixed and permeabilized using the BD Pharmagen™ Transcription Factor Buffer Set (BD Biosciences, 562574) according to the manufacturer’s instructions and stained with a fluorochrome-conjugated monoclonal antibody targeting the intracellular marker FoxP3 and 1:10 Brilliant Stain Buffer (BD Biosciences, 563794). All flow cytometry data were acquired using the BD FACSymphony™ A3 Cell Analyzer (BD Biosciences) and analyzed using FlowJo (v10, FlowJo LLC). Mouse spleen and lymph nodes were used as positive controls for all markers.

### Statistical analysis

All statistical analysis were performed on GraphPad Prism 9 (GraphPad, Inc). 2-way ANOVA tests with Tukey multiple comparisons *post-hoc* was used to determine statistical significance. *p < 0.05, **p < 0.01, ***p < 0.001, ****p < 0.0001. Data is presented as Mean ± Standard error of mean.

## Results

### Surgical technique for procurement of mouse hindlimb

**Figure 1:**
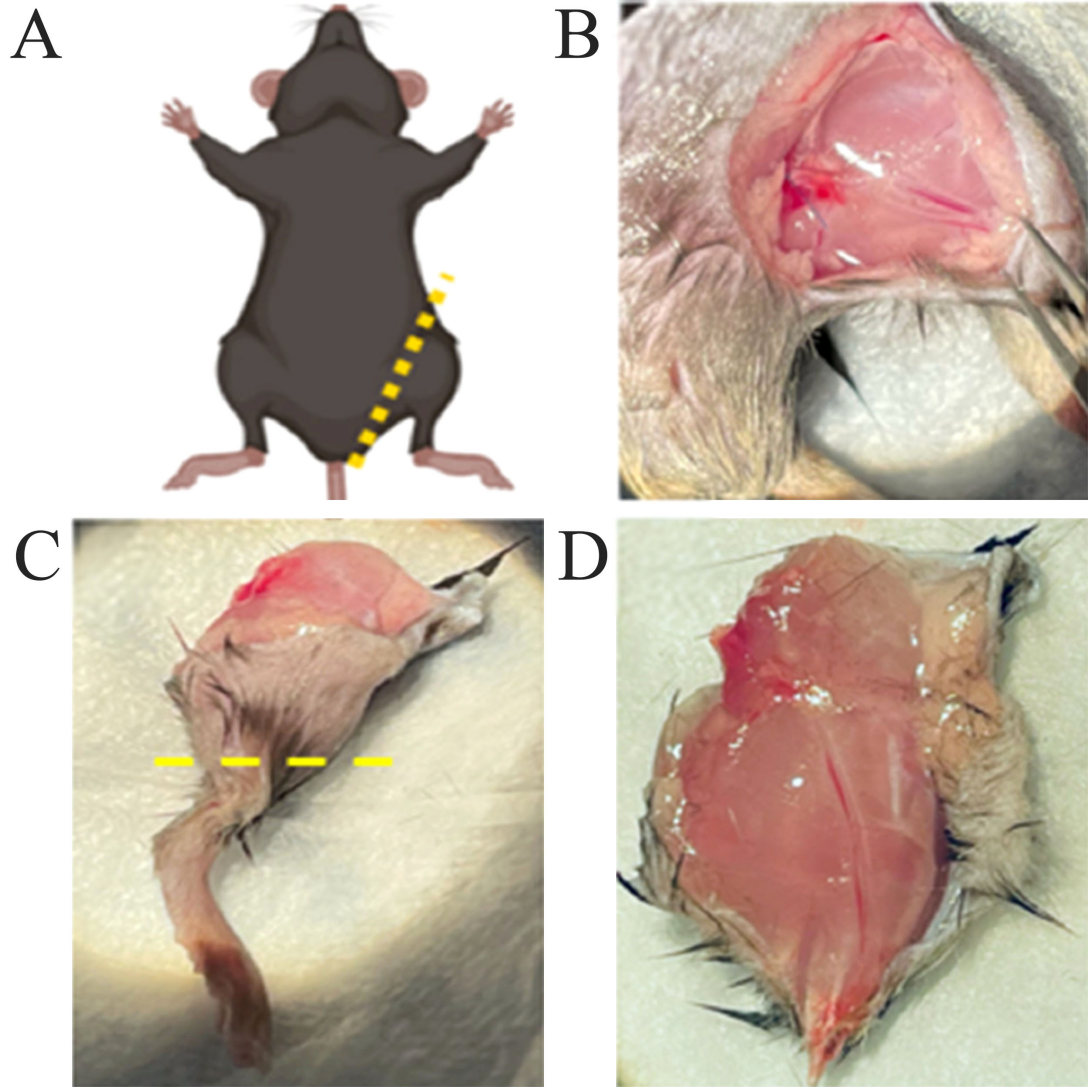
Procurement of mouse hindlimb. **(A)** Schematic illustrating the donor mouse in a supine position, with marking of the skin incision at the inguinal ligament level. **(B)** View of the exposed femoral vessels and underlying muscles after the incision was made. **(C)** Isolated hindlimb with marking of the transection at the knee level. **(D)** Isolated upper hindlimb after trimming of excess skin.

The procurement protocol was successful in isolating the upper hindlimb for subsequent transplantation into the recipient. The dissection images shown in **Figures 1A, B**, show the incision location at the inguinal ligament and exposure of underlying femoral vessels and muscles. **Figures 1C, D** display the isolated upper hindlimb following bone transection at the knee, with excess skin removed.

### Heterotopic transplantation

**Figure 2:**
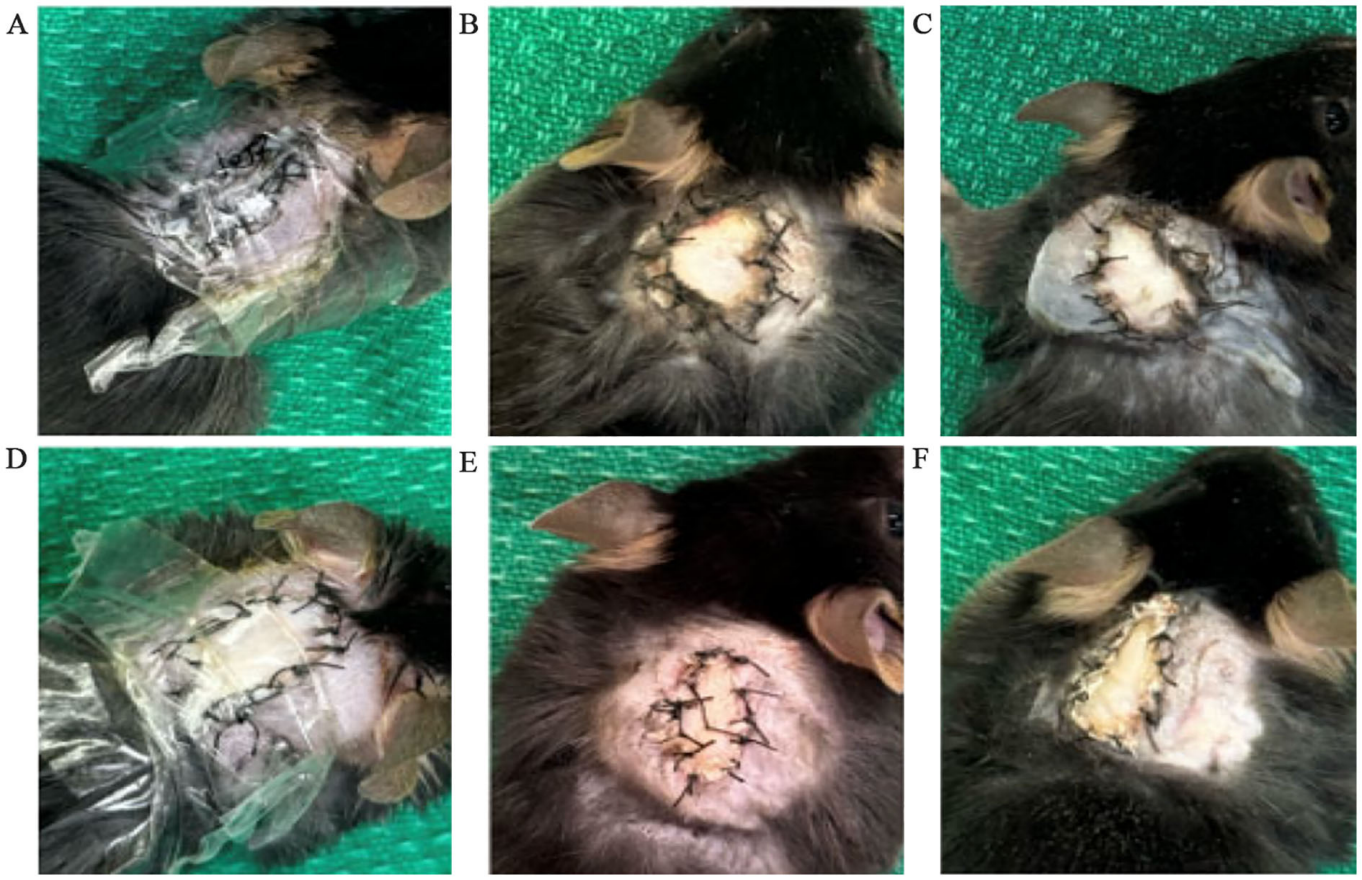
Condition of syngeneic and allogeneic grafts. **(A)** Syngeneic graft at post-operative day 0 (POD0) with a clear bandage applied. **(B)** Syngeneic graft at POD7 with the bandage removed. **(C)** Syngeneic graft at POD14 with the bandage removed. **(D)** Allogeneic graft at POD0 with a clear bandage applied. **(E)** Allogeneic graft at POD7 with the bandage removed. **(F)** Allogeneic graft at POD14 with the bandage applied.

The transplant procedure was successful in transplanting the graft into the dorsal upper thoracic region of the recipient mouse and securing the graft through the entire monitoring period. All except 1 out of 28 recipients survived the full 7- or 14-days monitoring period. One recipient was lost in the syngeneic 14-day monitoring period group due to reasons unrelated to the transplant procedure and was sacrificed. **Figures 2A–F** show that both syngeneic and allogeneic grafts were well maintained and were without any signs of scarring, necrosis, or external damage observed for the full duration of the monitoring period.

**Figure 3:**
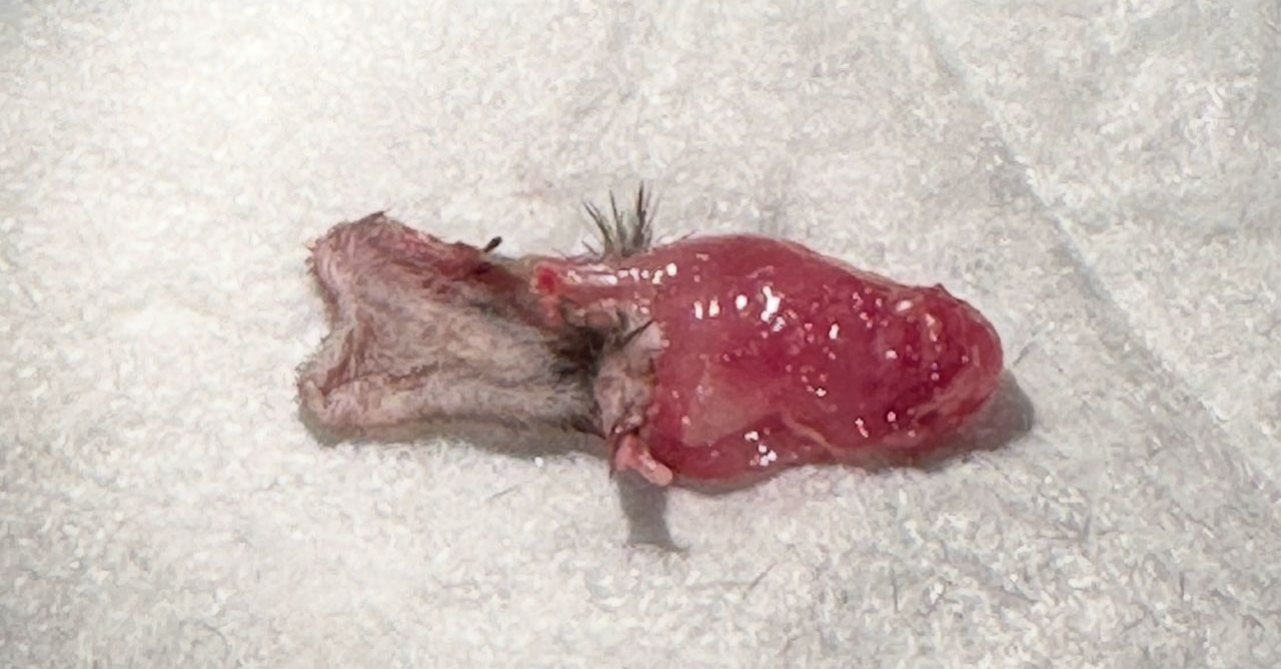
Vascularized composite allograft 14 days after transplantation. Left – skin. Right – muscle.

Recipient mice are sacrificed at the end of the monitoring period. The bandage and sutures were removed for graft procurement. Procured grafts were then separated into skin, muscle, and femur as shown in **Figure 3**.

### Histological analysis of syngeneic and allogeneic grafts

**Figure 4:**
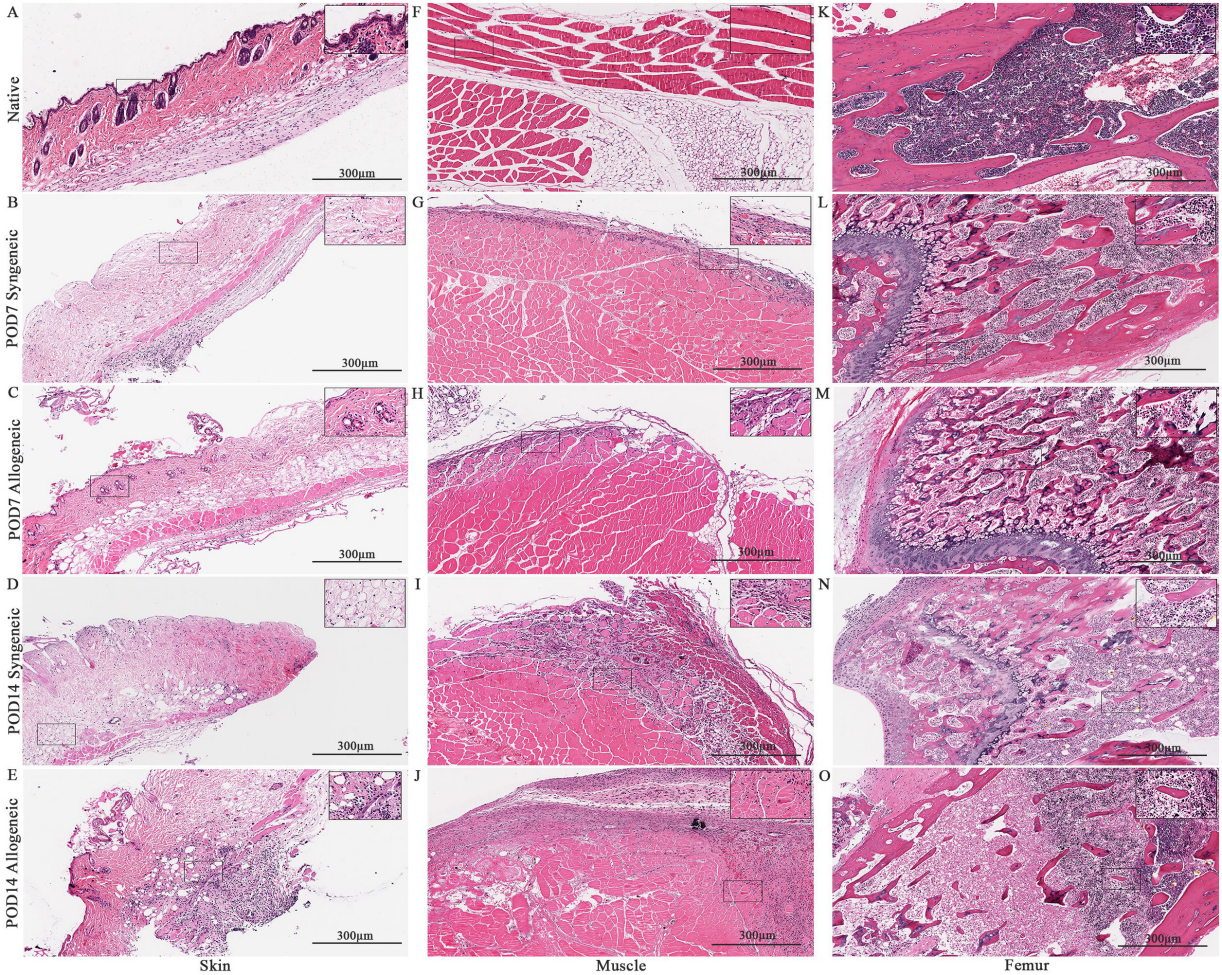
Histological analysis of native tissue, syngeneic and allogeneic vascularized composite allografts at POD7 and POD14 using Hematoxylin and Eosin (H&E). H&E stained native skin **(A)**, muscle **(F)**, and femur **(K)**. H&E stained syngeneic skin and allogeneic skin compared at POD7 **(B, C)** and POD14 **(D, E)**. H&E stained syngeneic muscle and allogeneic muscle compared at POD7 **(G, H)** and POD14 **(I, J)**. H&E stained syngeneic femur and allogeneic femur compared at POD7 **(L, M)** and POD14 **(N, O)**. Scale bar: 300 µm. n = 7 (POD7); n = 6 (POD14).

Isolated native and skin, muscle, and femur were stained with H&E to visualize the tissues on a microscopic level. The cellular content of syngeneic graft and allogeneic grafts at post-operative day 7 (POD7) and post-operative day 14 (POD14) were analyzed relative to native mouse hindlimb tissue and relative to each other. Generally, both syngeneic and allogeneic graft skin and muscle showed an increase in cellular content at POD7 and POD14 (**Figure 4**). Within the graft, the lowest cellular content was observed in the syngeneic graft skin at both POD7 and POD14 (**Figures 4B, D**). This was contrasted by the allogeneic graft skin, which showed relatively more cellular content at the subcutaneous level compared to native syngeneic graft skin (**Figure 4A**), particularly at POD14 (**Figure 4E**) but also at POD7 (**Figure 4C**). Additionally, vascular structures were also observed in the skin. The syngeneic muscle showed increased cellular content near the exterior of the tissue at POD7 (**Figure 4G**) relative to the native muscle (**Figure 4F**), with even more cellular content observed at POD14 (**Figure 4I**). This effect was observed in the allogeneic muscle more prominently at both POD7 and POD14 (**Figures 4H, J**). The syngeneic and allogeneic femurs showed fewer notable results at POD7 (**Figures 4L, M**). The femur at POD14, however, showed a slight decrease in cellular content in both the syngeneic and allogeneic models (**Figures 4M, O**), in comparison to the native femur (**Figure 4K**). Overall, H&E staining of the syngeneic and allogeneic graft skin and muscle showed various degrees of cell infiltration, with the allogeneic skin showing subclinical levels of rejection characterized by mild dermal infiltration.

### Characterization and quantification of immune cell population within syngeneic and allogeneic grafts

**Figure 5:**
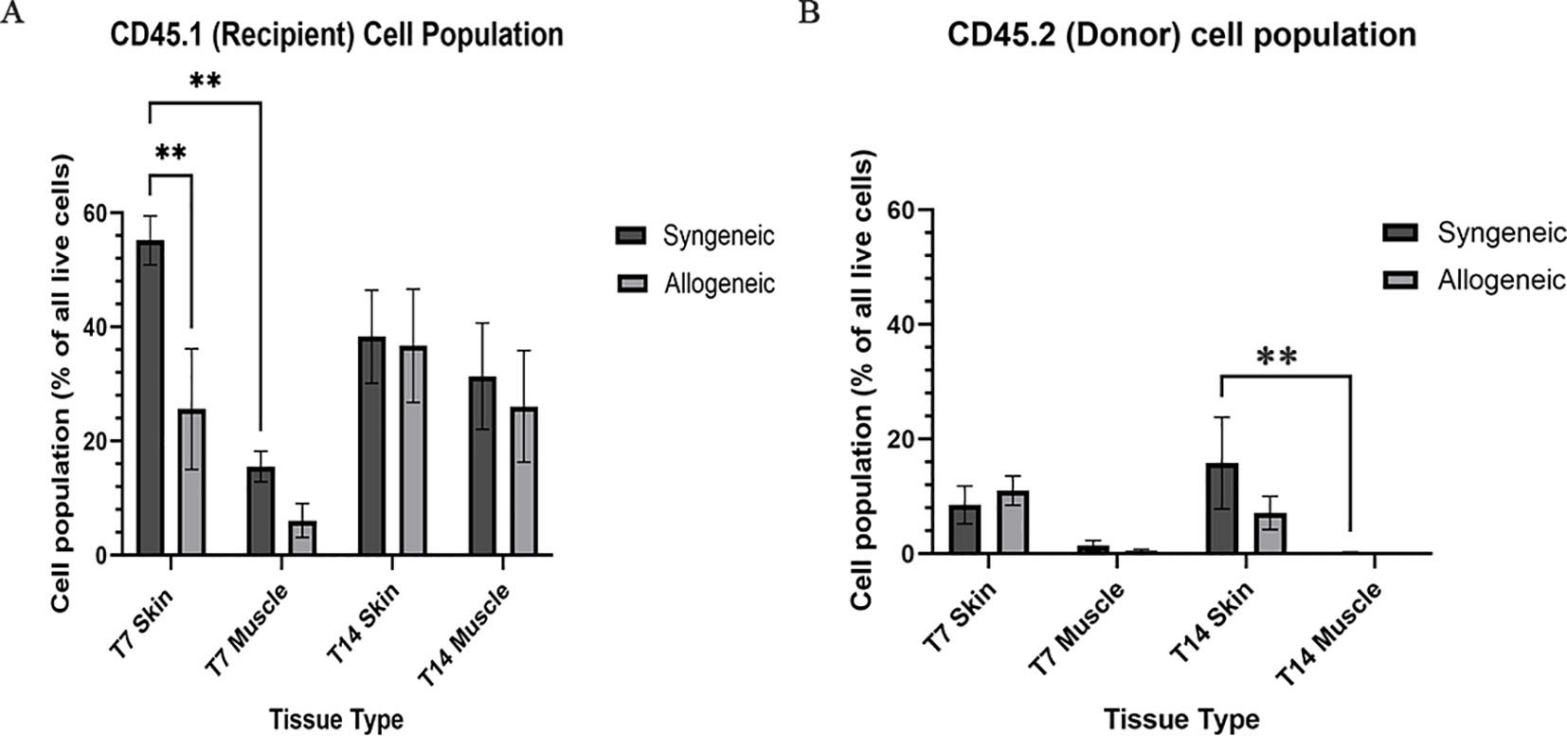
Quantification of total recipient (CD45.1) and donor (CD45.2) cell population in syngeneic and allogeneic vascular composite allografts. **(A)** Significantly more recipient cells were observed in syngeneic skin at POD7 (55.21 ± 4.28) versus allogeneic skin at POD7 (25.61 ± 12.50) and syngeneic skin at POD14 (38.30 ± 8.17). **(B)** Significantly more donor cells were observed in syngeneic skin (15.85 ± 8.00) relative to the syngeneic muscle (0.2290 ± 0.12) was observed at POD14. Cell populations were measured as a percentage of total live cells. Fresh tissue samples were processed and fluorescently labelled prior to detection by flow cytometry using BD FACSymphony A3 Cell Analyzer. n = 6 (syngeneic POD14); n = 7 (syngeneic/allogeneic POD7 and allogeneic POD14). Statistical analysis: 2-way ANOVA with Tukey multiple comparisons *post-hoc* (**p < 0.01).

**Figure 6:**
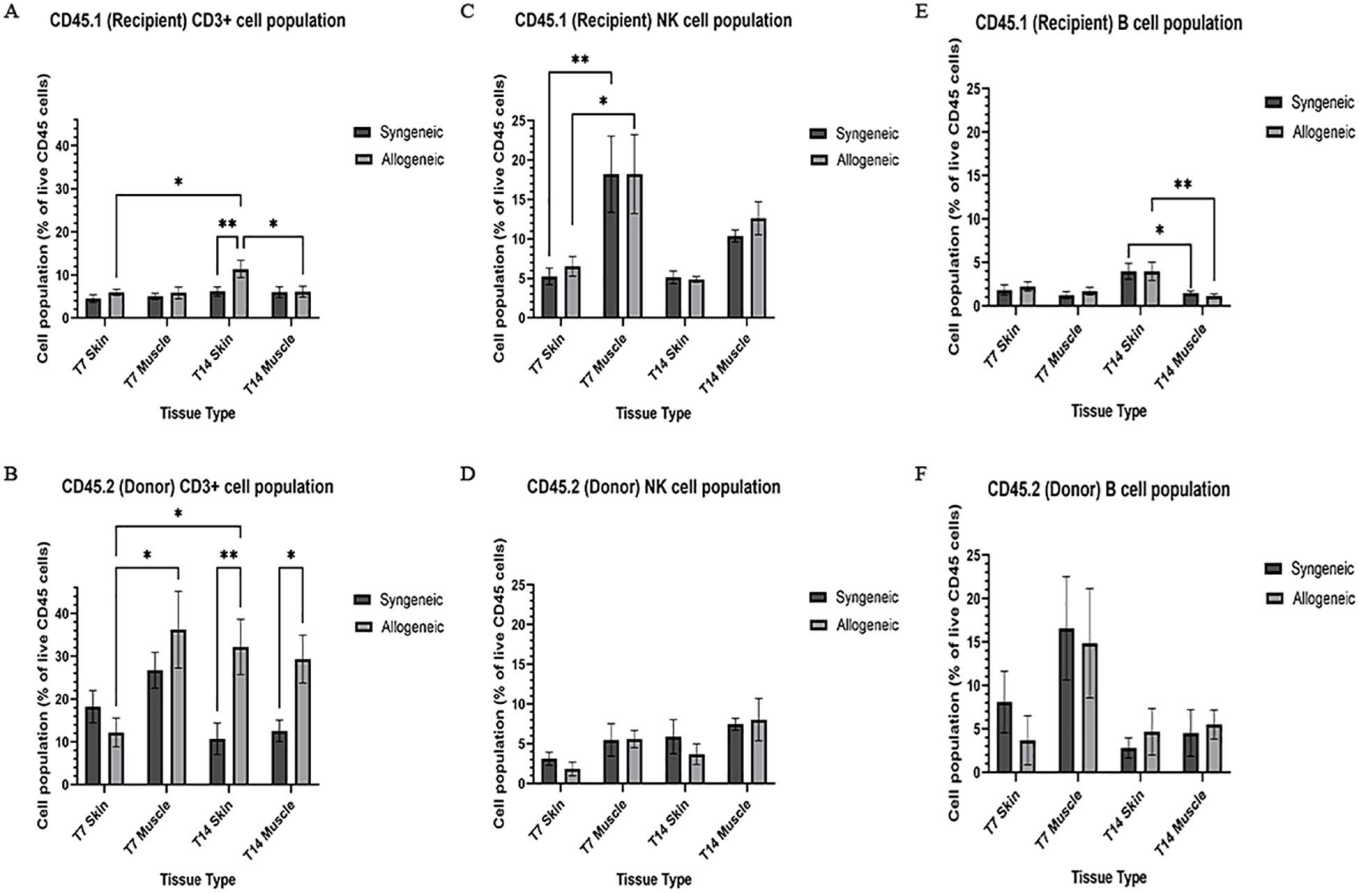
Quantification of recipient (CD45.1) and donor (CD45.2) CD3+ T cell, NK cell, and B cell populations. Cell population as a percentage of total recipient cell population or total donor population for CD3+ T cell **(A, B)**, NK cell **(C, D)**, and B cell **(E, F)** populations in syngeneic and allogeneic skin and muscle at POD7 and POD14. Fresh tissue samples were processed and fluorescently labelled prior to detection by flow cytometry using BD FACSymphony A3 Cell Analyzer. n = 6 (syngeneic POD14); n = 7 (syngeneic/allogeneic POD7 and allogeneic POD14). Statistical analysis: 2-way ANOVA with Tukey multiple comparisons *post-hoc* (*p < 0.05, **p < 0.01).

**Figure 7:**
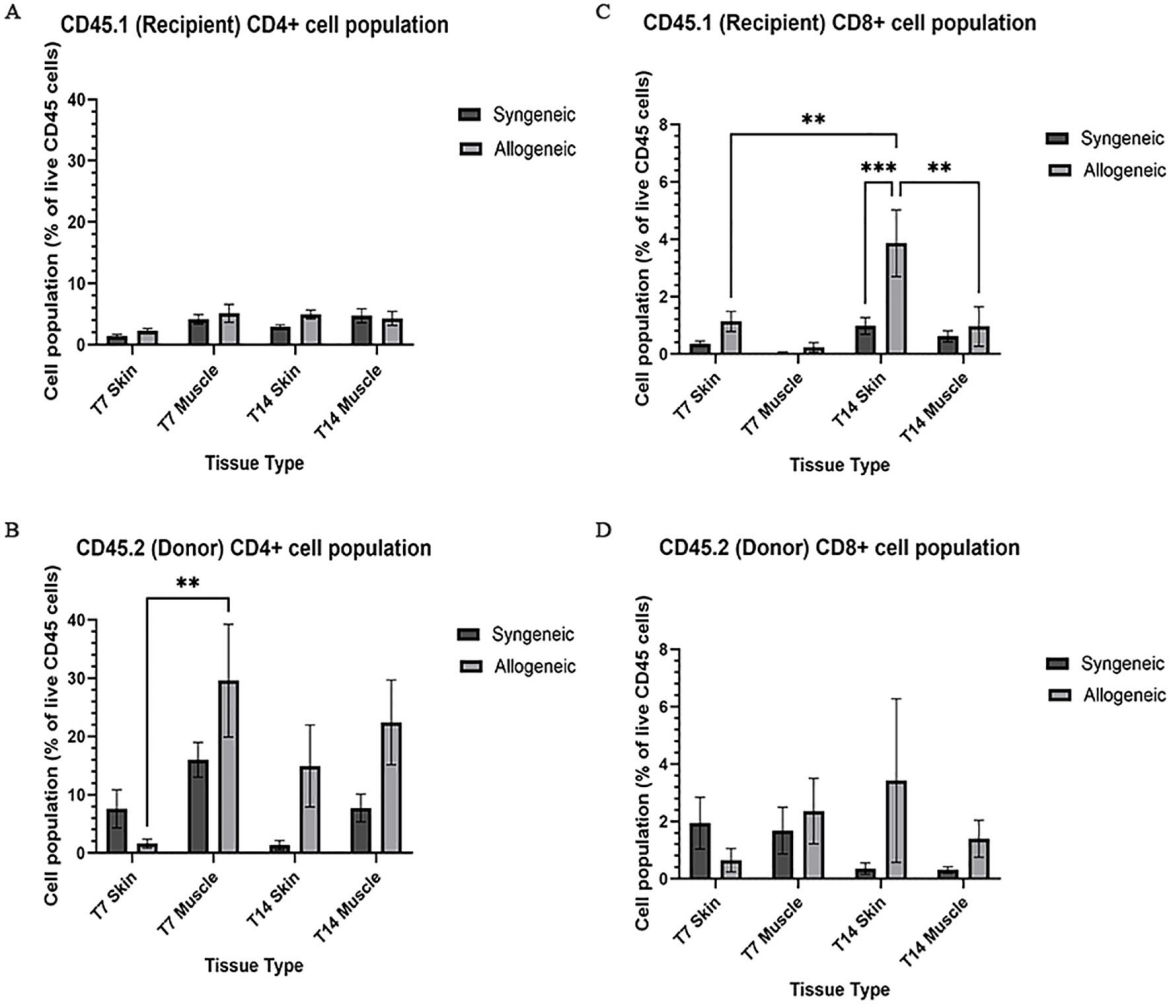
Quantification of recipient (CD45.1) and donor (CD45.2) CD4+ and CD8+ T cell populations. Cell population as a percentage of total recipient cell population or total donor population for CD4+ T cell **(A, B)** and CD8+ **(C, D)** populations in syngeneic and allogeneic skin and muscle at POD7 and POD14. Fresh tissue samples were processed and fluorescently labelled prior to detection by flow cytometry using BD FACSymphony A3 Cell Analyzer. n = 6 (syngeneic POD14); n = 7 (syngeneic/allogeneic POD7 and allogeneic POD14). Statistical analysis: 2-way ANOVA with Tukey multiple comparisons *post-hoc* (**p < 0.01, ***p < 0.001).

**Figure 8:**
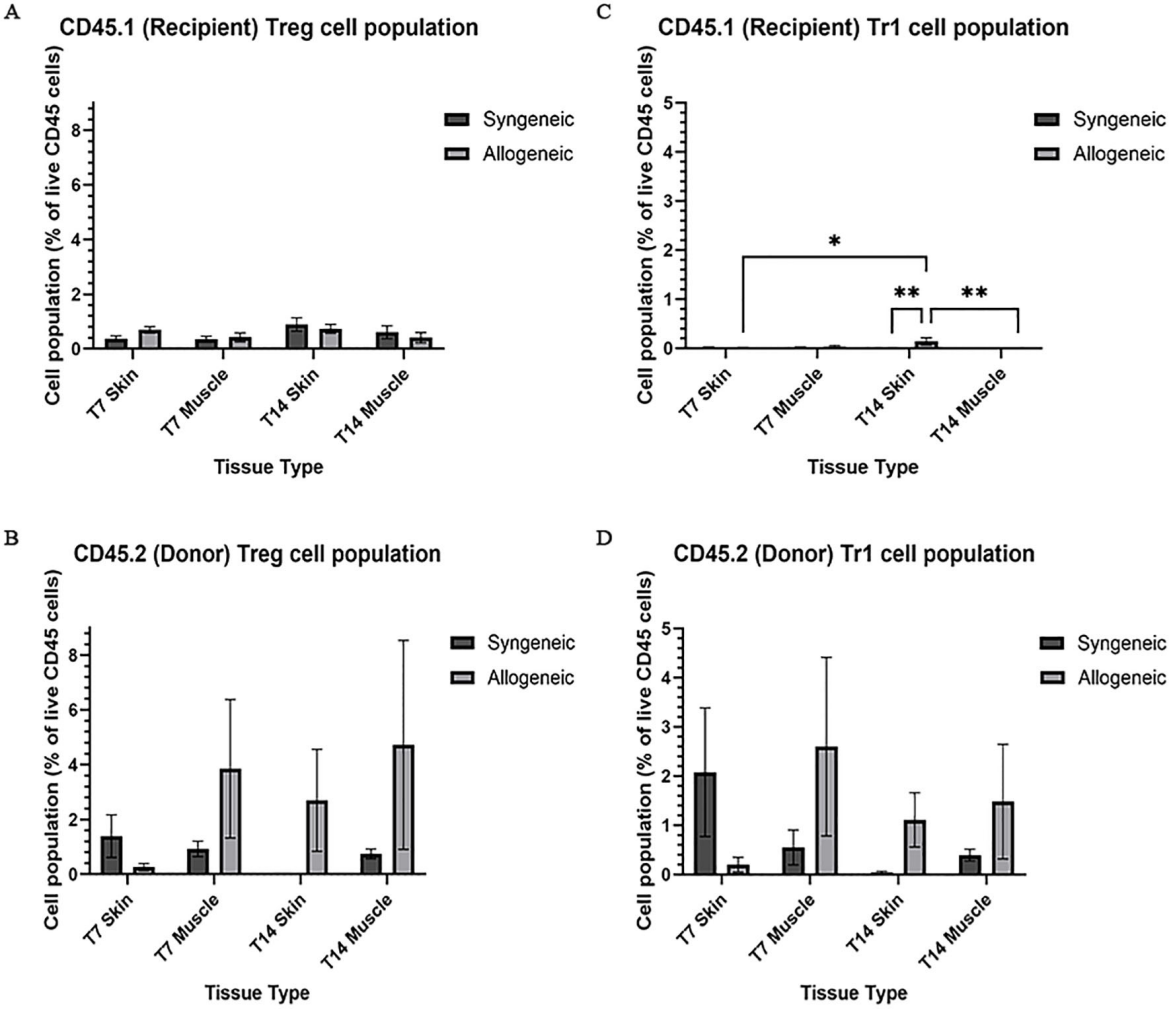
Quantification of recipient (CD45.1) and donor (CD45.2) CD4+ T cell subpopulations. Cell population as a percentage of total recipient cell population or total donor population for Treg **(A, B)** and Tr1 **(C, D)** populations in syngeneic and allogeneic skin and muscle at POD7 and POD14. Fresh tissue samples were processed and fluorescently labelled prior to detection by flow cytometry using BD FACSymphony A3 Cell Analyzer. n = 6 (syngeneic POD14); n = 7 (syngeneic/allogeneic POD7 and allogeneic POD14). Statistical analysis: 2-way ANOVA with Tukey multiple comparisons *post-hoc* (*p < 0.05, **p < 0.01).

**Figure 9:**
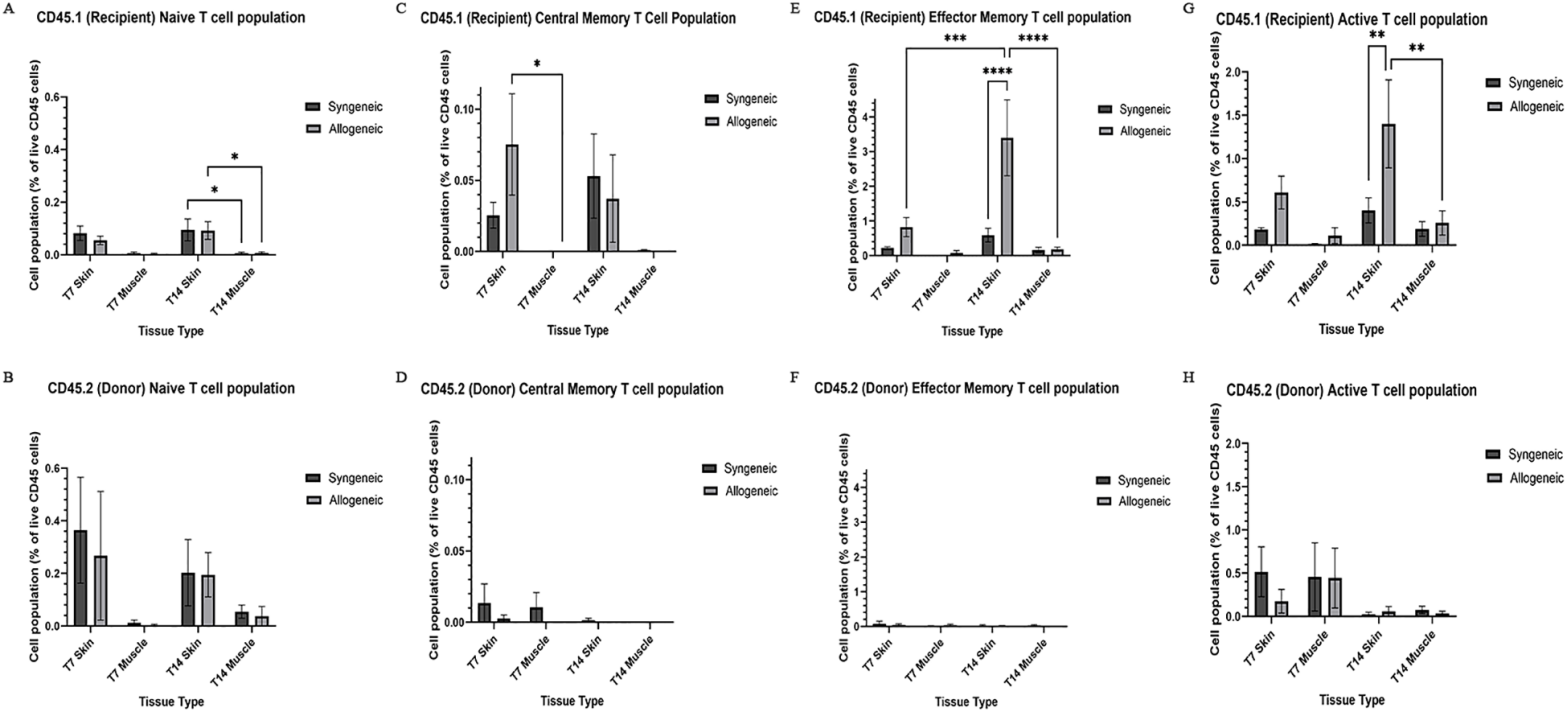
Quantification of recipient (CD45.1) and donor (CD45.2) CD8+ T cell subpopulations and active T cell subpopulations. Cell population as a percentage of total recipient cell population or total donor population for naïve T cell (CD44-, CD62L+) **(A, B)**, central memory T cell (CD44+, CD62L+) **(C, D)**, effector memory T cell (CD44+, CD62L-) **(E, F)**, and active T cell (CD8+, CD69+/CD25+) **(G, H)** populations in syngeneic and allogeneic skin and muscle at POD7 and POD14. Fresh tissue samples were processed and fluorescently labelled prior to detection by flow cytometry using BD FACSymphony A3 Cell Analyzer. n = 6 (syngeneic POD14); n = 7 (syngeneic/allogeneic POD7 and allogeneic POD14). Statistical analysis: 2-way ANOVA with Tukey multiple comparisons *post-hoc* (*p < 0.05, **p < 0.01, ***p < 0.001, ****p < 0.0001).

Immune cell populations were characterized by flow cytometry using a custom 16-colour panel. To generate a single-cell suspension from the skin and muscle, an optimized tissue dissociation protocol was applied, with spleen tissue digested via mechanical dissociation serving as a positive control (**Supplementary Figure 7**). Generally, the syngeneic skin at POD7 (55.21 ± 4.28) contained significantly more recipient-derived cell population relative to the allogeneic skin at POD7 (25.61 ± 12.50; p = 0.0089) and the syngeneic muscle at POD7 (15.55 ± 2.67 p = 0.0035) (**Figure 5A**). Overall, the tissues contained less donor-derived cells. The syngeneic skin at POD14 (15.85 ± 8.00) contained significantly more donor-derived cells than the syngeneic muscle at POD14 (0.2290 ± 0.12; p= 0.0091) (**Figure 5B**). The recipient-derived NK cell population in both syngeneic (18.20 ± 4.83) and allogeneic muscle at POD7 (18.22 ± 4.99) was observed to be significantly more than both syngeneic (5.260 ± 1.072; p = 0.0080) and allogeneic skin at POD7 (6.534 ± 1.413; p = 0.0193) (**Figure 6C**). No significant differences were observed in the corresponding donor-derived NK cell populations (**Figure 6D**). Both syngeneic (3.988 ± 0.917) and allogeneic (3.980 ± 1.052) skin showed a larger B cell population compared to both syngeneic (1.493 ± 0.220; p = 0.0466) and allogeneic muscle (1.133 ± 0.258; p = 0.0092) at POD14 (**Figure 6E**), while donor-derived B cells did not show significant variation (**Figure 6F**). Notably, there was a significant increase in both the recipient-derived (11.38 ± 2.01) 5.936 ± 0.716) and donor-derived (32.17 ± 6.45) T cell population in the allogeneic skin at POD14 compared to POD7 recipient (5.936 ± 0.716; p = 0.0138) and donor (12.18 ± 3.35; p = 0.0466) (**Figures 6A, B**). Additionally, the donor-derived CD3+ T cell population was observed to be larger in the allogeneic group skin (32.17 ± 6.45) and muscle (29.31 ± 5.62) compared to respective syngeneic groups in POD14 skin (10.73 ± 3.68; p = 0.079) and POD14 muscle (12.55 ± 2.54; p = 0.0351) (**Figure 6B**). Specifically, in the T cell populations, more donor-derived CD4+ T cells were observed in the allogeneic muscle (29.57 ± 9.66) compared to the allogeneic skin (1.619 ± 0.759; p = 0.0032) at POD7 (**Figure 7B**). No significant differences were observed in recipient CD4+ T cells (**Figure 7A**) or CD8+ donor T cells (**Figure 7D**). However, a significant increase in recipient-derived CD8+ T cells was observed in the allogeneic skin from POD7 (1.134 ± 0.350) to POD14 (3.860 ± 1.166; p = 0.0031), but not in the syngeneic skin (**Figure 7C**).Within the CD4+ T cell subpopulations, recipient-derived Tr1 cells have been observed to be significantly more in the allogeneic skin at POD14 (0.1389 ± 0.0726) relative to the syngeneic skin at POD14 (0.0002883 ± 0.0002883; p = 0.0015), as well as the allogeneic skin at POD7 (0.01359 ± 0.00355; p = 0.0133) (**Figure 8C**). Interestingly, no significant differences were observed in recipient Treg cells (**Figure 8A**), donor Treg cells (**Figure 8B**) or donor Tr1 cells (**Figure 8D**). Further, allogeneic skin at POD14 in recipient-derived naïve (0.092 ± 0.034) and effector memory T cells (3.4 ± 1.1) significantly increase in comparison to POD14 allogeneic muscle in recipient naïve (0.00657 ± 0.00463; p = 0.0318) and effector memory T cells (0.1841 ± 0.0608; p = < 0.0001) (**Figures 9A, E**). No significant differences were observed in donor naïve T cells (**Figure 9B**), recipient and donor central memory T cells (**Figures 9C, D**), or donor effector memory T cells (**Figure 9F**). A significant increase in active T cells was observed in allogeneic skin (1.4 ± 0.51) compared to allogeneic muscle (0.2581 ± 0.1340; p = 0.0022) at POD14 (**Figure 9G**), while donor active T cells did not differ significantly (**Figure 9H**).

### Temporal variation of immune and apoptotic markers in syngeneic and allogeneic skin and muscle grafts

**Figure 10:**
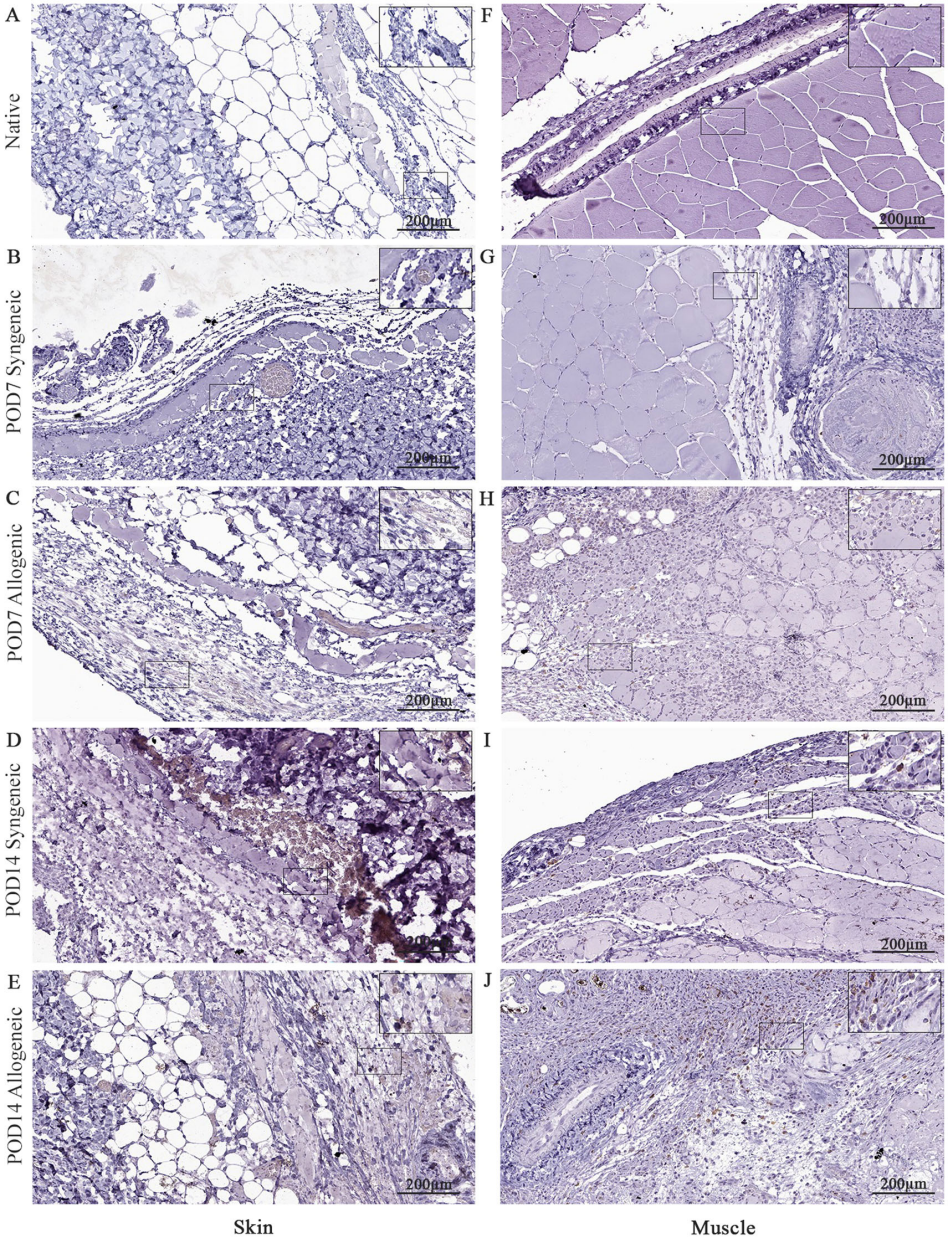
Immunohistochemical analysis of native tissue, syngeneic and allogeneic vascularized composite allografts at POD7 and POD14 using CD8 antibody. Native skin **(A)** and muscle **(F)**. Syngeneic skin and allogeneic skin compared at POD7 **(B, C)** and POD14 **(D, E)**. Syngeneic muscle and allogeneic muscle compared at POD7 **(G, H)** and POD14 **(I, J)**. Scale bar: 200 µm. n = 3.

**Figure 11:**
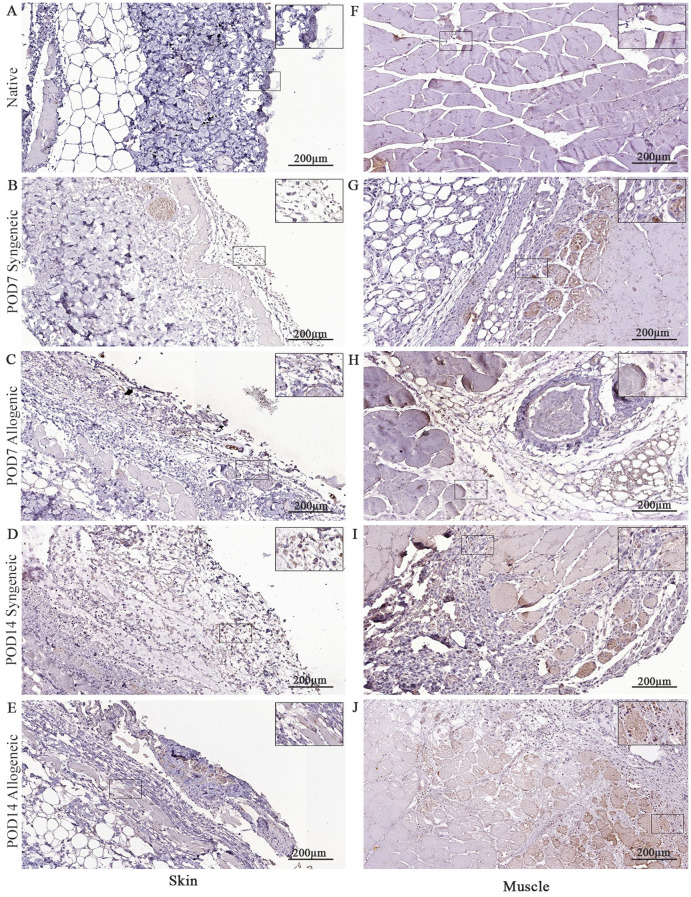
Immunohistochemical analysis of native tissue, syngeneic and allogeneic vascularized composite allografts at POD7 and POD14 using caspase-3 antibody. Native skin **(A)** and muscle **(F)**. Syngeneic skin and allogeneic skin compared at POD7 **(B, C)** and POD14 **(D, E)**. Syngeneic muscle and allogeneic muscle compared at POD7 **(G, H)** and POD14 **(I, J)**. Scale bar: 200 µm. n = 3.

**Figure 12:**
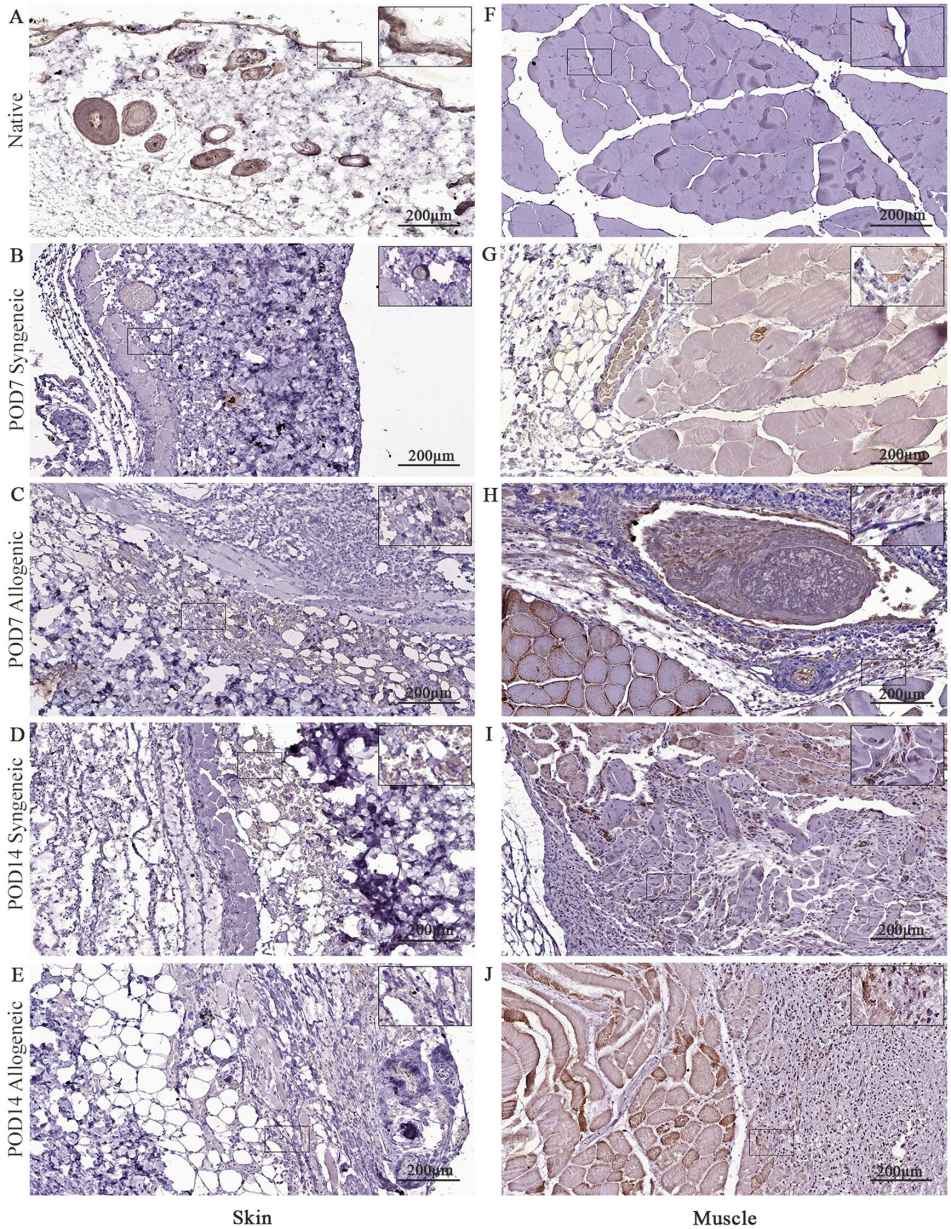
Immunohistochemical analysis of native tissue, syngeneic and allogeneic vascularized composite allografts at POD7 and POD14 using CD31 antibody. Native skin **(A)** and muscle **(F)**. Syngeneic skin and allogeneic skin compared at POD7 **(B, C)** and POD14 **(D, E)**. Syngeneic muscle and allogeneic muscle compared at POD7 **(G, H)** and POD14 **(I, J)**. Scale bar: 200 µm. n = 3.

Native and grafted skin and muscle samples were isolated and stained for CD8, caspase-3, and CD31 to assess inflammatory T-cell presence, apoptosis, and tissue vascularization, respectively. In general, all three markers exhibited a distinct increase at POD14 in both syngeneic and allogeneic skin and muscle samples compared to POD7 (**Figures 10**–**12**). Interestingly, at POD7, CD8-positive cells were absent from the skin grafts (**Figures 10B, C**) and minimally present in the muscle (**Figures 10G, H**); however, by POD14, CD8-positive cells were clearly detectable in both skin (**Figures 10D, E**) and in muscle (**Figures 10I, J**). In addition, caspase-3 staining revealed an evident increase in apoptotic cells at POD14 compared to POD7 in both syngeneic and allogeneic skin and muscle tissues (**Figures 11D, E, I, J**).

### Graft revascularization

CD31 was also analyzed in our study to assess tissue revascularization in both syngeneic and allogeneic grafts. There was no clear difference observed in CD31 expression between syngeneic and allogeneic skin samples at both POD7 (**Figures 12B, C**) and POD14 (**Figures 12D, E**). At POD7, CD31 expression was particularly pronounced in the allogeneic muscle (**Figure 12H**), and by POD14, both syngeneic (**Figure 12I**) and allogeneic muscle (**Figure 12J**) tissues demonstrated substantial levels of CD31, revealing a distinct increase in revascularization. This is noteworthy, given that our model utilizes non-vascularized grafts, which rely on neovascularization from the recipient for survival. The substantial levels of CD31 in both syngeneic and allogeneic muscle tissues at POD14 suggests that recipient-derived endothelial cells are actively contributing to the formation of new blood vessels within the transplanted grafts.

### Differential apoptosis in syngeneic and allogeneic skin and muscle grafts at POD7 and POD14

**Figure 13:**
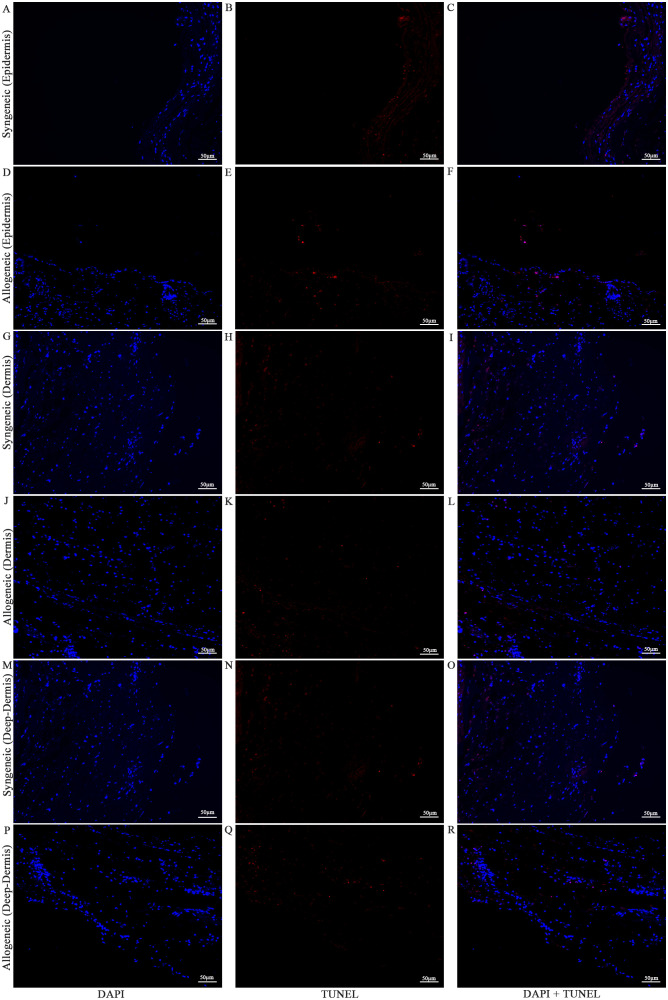
Apoptotic staining of syngeneic and allogeneic vascularized composite skin allografts at day 7 using Terminal Deoxynucleotidyl Transferase dUTP Nick End Labeling (TUNEL). Epidermis of syngeneic and allogeneic skin at POD7 was compared and stained with DAPI only **(A, D)**, TUNEL only **(B, E)** and DAPI + TUNEL **(C, F)**. Dermis of syngeneic and allogeneic skin at POD7 was compared and stained with DAPI only **(G, J)**, TUNEL only **(H, K)** and DAPI + TUNEL **(I, L)**. Deep-dermis of syngeneic and allogeneic skin at POD7 was compared and stained with DAPI only **(M, P)**, TUNEL only **(N, Q)** and DAPI + TUNEL **(O, R)**. Scale bar: 50 µm. n = 3.

**Figure 14:**
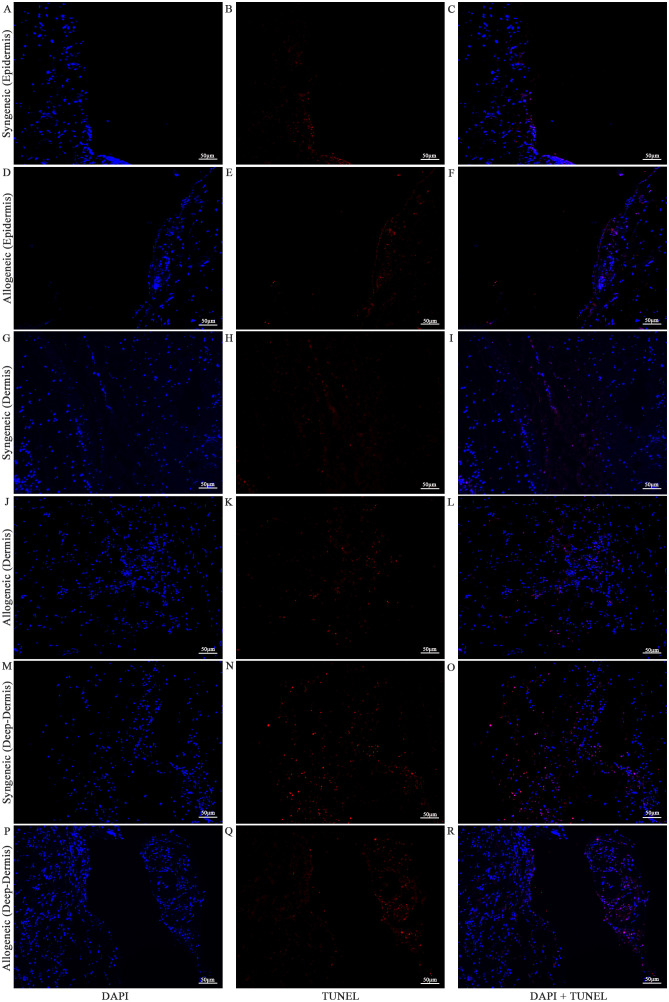
Apoptotic staining of syngeneic and allogeneic vascularized composite skin allografts at day 14 using Terminal Deoxynucleotidyl Transferase dUTP Nick End Labeling (TUNEL). Epidermis of syngeneic and allogeneic skin at POD14 was compared and stained with DAPI only **(A, D)**, TUNEL only **(B, E)** and DAPI + TUNEL **(C, F)**. Dermis of syngeneic and allogeneic skin at POD14 was compared and stained with DAPI only **(G, J)**, TUNEL only **(H, K)** and DAPI + TUNEL **(I, L)**. Deep-dermis of syngeneic and allogeneic skin at POD14 was compared and stained with DAPI only **(M, P)**, TUNEL only **(N, Q)** and DAPI + TUNEL **(O, R)**. Scale bar: 50 µm. n = 3.

**Figure 15:**
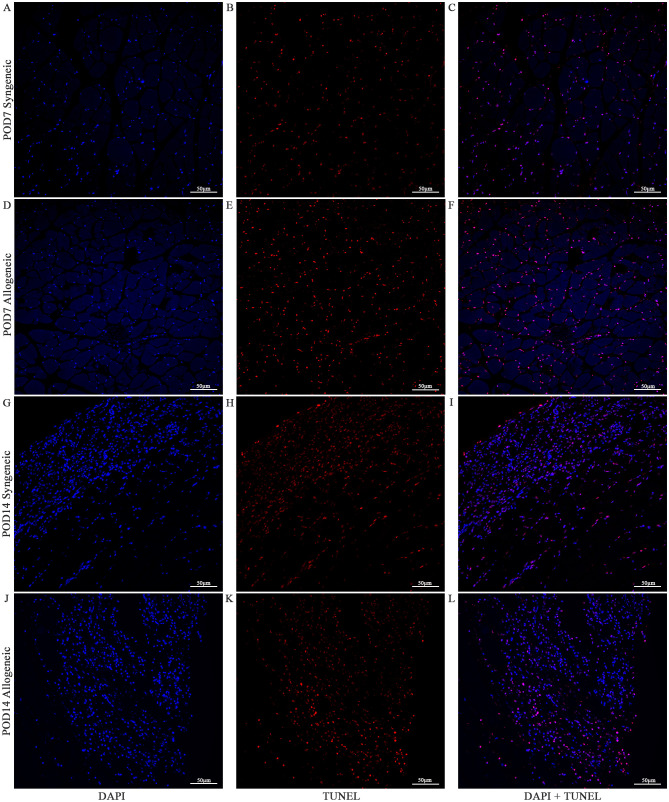
Apoptotic Staining of syngeneic and allogeneic vascularized composite muscle allografts at day 7 and day 14 using Terminal Deoxynucleotidyl Transferase dUTP Nick End Labeling (TUNEL). Syngeneic and allogeneic muscle at POD7 was compared and stained with DAPI only **(A, D)**, TUNEL only **(B, E)** and DAPI + TUNEL **(C, F)**. Syngeneic and allogeneic muscle at POD14 was compared and stained with DAPI only **(G, J)**, TUNEL only **(H, K)** and DAPI + TUNEL **(I, L)**. Scale bar: 50 µm. n = 3.

Cell viability in the skin and muscle was assessed using TUNEL staining to detect apoptotic cells (**Figures 13**–**15** **).** Skin sections were analyzed at multiple anatomical layers, including the epidermis, dermis, and deep dermis, to evaluate apoptosis. A greater number of TUNEL-positive cells were observed in the epidermis (**Figures 13F**, **14F**) and dermis (**Figures 13L**, **14L**) of allogeneic skin samples compared to syngeneic skin samples at both POD7 and POD14. In contrast, no clear differences in the number of TUNEL-positive cells were detected between syngeneic and allogeneic deep dermal skin samples at either time point (**Figures 13O, R**; **14O, R**). Additionally, a greater number of TUNEL-positive cells were observed in the allogeneic muscle at POD7 (**Figure 15F**) compared to syngeneic muscle (**Figure 15C**), whereas at POD14, there is no clear difference between the syngeneic and allogeneic samples (**Figures 15I, L**).

## Discussion

In this study, we established a non-vascularized mouse heterotopic partial hindlimb transplant model to investigate immune cell dynamics during early acute rejection. Using a custom 16-color flow cytometry panel and histological analysis, we characterized immune cell infiltrates in syngeneic and allogeneic grafts. Our findings reveal significant increases in CD8+ T cells and Tr1 cells in allogeneic skin at POD14, paralleled with increased cell apoptosis observed. Histological analysis further demonstrated subclinical rejection in allogeneic grafts, characterized by mild dermal infiltration and increased cellular content. Notably, we observed an increase in CD31 positivity in syngeneic and allogeneic muscle at POD14 despite not having performed any anastomosis, suggesting the possibility of neovascularization within transplanted grafts. These results provide new insights into the immune mechanisms driving acute rejection and highlight potential therapeutic targets for tolerance induction.

Currently, both rodents and porcine models have been utilized in VCA studies, with large animal models offering clinical translatability and small animal models, particularly mice, being well established for immunological studies. While existing hindlimb transplant models in mice vary in graft location and vascularization (20, 21), the heterotopic hindlimb transplant model used in this study presents a distinctive approach. This model is particularly valuable due to its ability to assess immune responses during the acute rejection phase at critical monitoring time points of 7 and 14 days. Unlike prior models, this study employed a congenic strain system for both syngeneic and allogeneic grafts, allowing precise tracking of immune cell origin using the CD45.1 and CD45.2 markers, providing a clear distinction between donor-derived and recipient-derived cells.

Our model distinguishes itself through the observation of tissue-specific immune responses within the VCA setting, with notable differences in immune cell infiltration patterns across skin, muscle, and bone compartments. At POD14, histological and immunophenotypic analyses revealed dense infiltration of CD8+ T cells and B cells in skin, consistent with its high immunogenicity attributed to abundant antigen presenting cells and elevated MHC expression. In contrast, muscle demonstrated moderate immune cell infiltration, suggesting a comparatively immune-privileged environment, potentially mediated by its lower baseline MHC expression and anti-inflammatory environment. Bone tissue exhibited limited immune cell infiltration; however, given its hematopoietic capacity, it likely contributes to systemic immune regulation rather than serving as a primary site of rejection. These findings emphasize the importance of recognizing and addressing the unique immunological vulnerabilities of each tissue type in VCA. Specifically, skin may require intensified or localized immunosuppressive strategies to mitigate its heightened immune reactivity, while muscle may benefit from tolerance-promoting or regenerative approaches that preserve its function without excessive immunosuppression. The immunoregulatory properties of bone also present opportunities for systemic immune modulation through bone marrow-targeted therapies. Collectively, this data highlights the necessity of tissue-specific immunosuppressive protocols and provide a foundation for the development of tailored, tissue-directed tolerance-induction strategies aimed at improving long-term VCA outcomes. A deeper understanding of tissue-specific immunity could inform both clinical decision-making and the development of localized tolerance-induction therapies in VCA. A deeper understanding of tissue-specific immunity could inform both clinical decision-making and the development of localized tolerance-induction therapies in VCA.

While the heterotopic, non-vascularized design of our model does not fully replicate the anatomical and vascular features of clinical VCA procedures such as limb or facial transplants, it offers distinct advantages for mechanistic studies by eliminating technical variability introduced by vascular anastomosis. This model provides a controlled platform for investigating early immune responses and tissue-specific rejection patterns, which are highly relevant to clinical VCA, where skin and muscle respond differently to immune challenges. Specifically, this study observed robust immune cell infiltration and subclinical rejection in the skin, while muscle tissue exhibited a more limited immune response, consistent with its lower MHC expression and reduced vascularization. These findings highlight the importance of developing targeted immunosuppressive strategies based on the immunological characteristics of specific tissue types.

When examining the immune cell populations, we observed a significantly higher recipient-derived NK cell population in both the syngeneic and allogeneic muscle at POD7 relative to the skin. NK cells also display both inflammatory and regulatory functions in immune responses and have roles in promoting muscle regeneration through IFN-γ production (22). NK cells are important mediators in the cellular inflammation following acute muscle injury, and could be the cause of the increase in NK cell population in the muscle (23). However, the exact functions of the infiltrating NK cells we observed in the grafts need further characterization. We also observed the highest recipient-derived B cell population in the syngeneic and allogeneic skin at POD14.

The elevated B cell populations in allogeneic skin at POD14 underscore the complexity of immune responses in VCA. While B cells are traditionally studied for their role in antibody-mediated rejection through donor-specific antibodies (DSA) production (24), our findings suggest that B cells may also contribute to acute rejection through antigen presentation and T cell recruitment, as indicated by the significant increase in recipient-derived T cell populations in allogeneic grafts. This supports recent studies showing that B cells can function as professional antigen-presenting cells (APCs) and interact with other immune cells such as T cells (25). The role of B cells in VCA remains poorly understood, and further research is needed to determine whether these cells are donor-specific and whether they exert pathogenic or protective functions in graft rejection. Notably, we observed overall higher percentages of donor B cells and CD4+ T cells in allogeneic graft tissues, which are both involved in Graft versus Host Disease (GvHD) pathogenesis (26, 27). While acute GvHD occurs 2–4 weeks post-transplant in humans, it occurs within 7–14 days within mouse models (28), suggesting a potential connection between GvHD and VCA, warranting further exploration in future studies. Additionally, the role of B cells in VCA warrants further investigation, particularly their potential as therapeutic targets for modulating immune responses.

The central role of T cells in acute rejection is well-established, and our findings support previous studies highlighting the importance of CD8+ cytotoxic T cells in mediating graft damage through direct cytotoxicity and cytokine release (29, 30). We observed a significant increase in recipient-derived CD8+ T cells in allogeneic skin at POD14, along with elevated populations of naïve, effector memory, and active T cells. Immunohistochemistry further revealed a correlation between increased CD8+ positivity and apoptotic cells in allogeneic skin and muscle at POD14. These findings suggest that CD8+ T cells drive the early immune response against allogeneic grafts, even under immunosuppression with tacrolimus. However, the concurrent increase in Tr1 cells in allogeneic skin at POD14 indicates a potential regulatory mechanism that mitigates the cytotoxic effects of CD8+ T cells, maintaining subclinical rejection levels. Tr1 cells, known for their immunosuppressive functions, secrete anti-inflammatory cytokines such as IL-10 and TGF-β, which inhibit pro-inflammatory Th1 and Th17 responses (31–33). This balance between effector and regulatory T cells may be critical for maintaining graft tolerance and preventing rejection.

The significant increase in Tr1 cells in allogeneic grafts suggest that these cells may play a role in modulating immune responses and potentially promoting graft tolerance. Unlike conventional Tregs, which require cell-to-cell contact for suppression, Tr1 cells exert their effects primarily through cytokine secretion, making them an intriguing target for further investigation in therapeutic applications (31, 32). While the use of Tr1 cells in tolerance induction has been explored in islet transplantation, their role in VCA remains less understood. The potential for recipient-derived Tr1 cells to be expanded and introduced into grafts to promote tolerance could be an avenue for future research, although functional validation through studies such as adoptive transfer or cytokine blockade will be necessary to confirm their efficacy in this context. Future studies should also explore how Tr1 cells interact with other immune cells, including CD8+ T cells, and evaluate their long-term impact on graft survival.

Clinically, our findings highlight the need for tailored immunosuppressive strategies that consider the unique immunological properties of VCA grafts. By targeting specific immune cell populations, such as Tr1 cells, it may be possible to reduce dependence on broad-spectrum immunosuppression and its associated side effects, such as opportunistic infections and malignancies (3–5). This approach has the potential to significantly enhance the quality of life for VCA recipients and improve long-term graft outcomes. Additionally, developing biomarkers to monitor Tr1 cell activity and CD8+ T cell responses could enable personalized immunosuppressive regimens, optimizing graft outcomes while minimizing adverse effects.

While this model offers valuable insights into acute immune responses in VCA, certain limitations should be considered. The absence of vascular anastomosis in the heterotopic transplant model means it does not fully replicate the full complexity of clinical vascularized transplants, which is a limitation, but it also provides a controlled environment to study early immune dynamics without the added variability of vascular surgery. Additionally, the short postoperative monitoring periods focus on the acute rejection phase and do not capture the potential long-term, chronic rejection responses, which remain an important avenue for future exploration. The use of tacrolimus in both syngeneic and allogeneic groups may also obscure baseline immunological differences, highlighting the need for further studies with varying immunosuppressive regimens to better understand immune tolerance mechanisms. Furthermore, while the study design with sacrifices at POD7 and POD14 omits a longitudinal analysis, it provides critical snapshots of immune responses during the acute phase, offering foundational insights into future work. While immunohistochemistry was conducted in a blind manner, other analysis, such as flow cytometry, were not blinded, though the overall robustness of the findings supports their validity. Despite these limitations, the unique approach taken in this study – particularly the combination of a non-vascularized heterotopic model with tissue-specific immune response analysis – provides valuable insights into VCA immune dynamics and offers a strong foundation for the development of more targeted, personalized immunosuppressive therapies in future studies.

Overall, the findings in this study present a proof-of-concept mouse heterotopic partial hindlimb transplant model and characterizes immune cell infiltrates within the syngeneic and allogeneic grafts at POD7 and POD14. Significant changes in various recipient-derived and donor-derived immune cell populations were observed, including increased recipient CD8+ cytotoxic T cell subpopulations and Tr1 cells within the tolerant, allogeneic skin at POD14. By examining the condition of the grafts as well as the immune cell populations within, these findings highlight key cell populations that warrant further investigation to unravel the underlying cellular mechanisms driving acute rejection in VCA. Moreover, these results support the potential use of Tr1 cells in cell therapy for VCA tolerance induction. The insights gained from this study provide valuable information for understanding and mitigating acute rejection, while promoting long-term graft acceptance through targeted immunosuppressive strategies.

## Data Availability

The original contributions presented in the study are included in the article/**Supplementary Material**. Further inquiries can be directed to the corresponding authors.
