# Supplementary material for: Evaluating the immunogenicity of a mouse partial hindlimb for composite allotransplantation
Source: Front Immunol. 2025 Jun 18;16:1595319. doi: 10.3389/fimmu.2025.1595319 (PMC12213382; doi:10.3389/fimmu.2025.1595319)
Supplement: Supplementary file 1 [file DataSheet1.docx]

Supplementary Material

# Supplementary Tables

| Dilutions | 1:30 | 1:100 | 1:300 | 1:900 | 1:2700 |
| --- | --- | --- | --- | --- | --- |
| CD45.2-BUV395 | 24.5 | 21.3 | 9.79 | 4.08 | 3.27 |
| CD45.1-BUV395 | 27.2 | 13.0 | 5.93 | 3.02 | 2.44 |
| CD3-BV650 | 6.39 | 8.59 | 8.91 | 4.61 | 8.91 |
| CD4-BV480 | 26.8 | 28.5 | 34.6 | 19.0 | 11.1 |
| CD8a-APC-eF780 | 91.4 | 120 | 105 | 41.0 | 45.1 |
| CD25-PE | 22.4 | 28.5 | 27.4 | 12.4 | 9.45 |
| CD44-FITC | 8.12 | 14.1 | 13.9 | 14.4 | 12.9 |
| CD62L-PE-CF594 | 7.30 | 8.46 | 6.37 | 6.73 | 6.3 |
| CD127-BUV737 | 7.80 | 8.59 | 4.87 | 2.90 | 1.88 |
| CD69-BB700 | 4.65 | 6.45 | 7.50 | 5.82 | 4.99 |
| CD49b-BV421 | 36.5 | 38.2 | 76.0 | 74.0 | 56.3 |
| LAG3-BUV615 | 1.71 | 2.44 | 3.22 | 3.33 | 3.11 |
| NK1.1-BV711 | 6.19 | 6.18 | 6.14 | 5.26 | 5.38 |
| CD19-AF700 | 18.7 | 30.0 | 30.5 | 30.3 | 19.3 |
| Foxp3-F660 | 30.0 | 33.2 | 50.2 | 31.5 | 10.9 |

## Supplementary Table 1. Stain Index Values of Antibody Titrations. Stain index values calculated from each antibody dilution are shown in order. A higher stain index indicates a higher resolution in the fluorescent signal. All data was acquired by flow cytometry using BD FACSymphony A3 Cell Analyzer and analyzed using FlowJo (v10, FlowJo LLC).

##
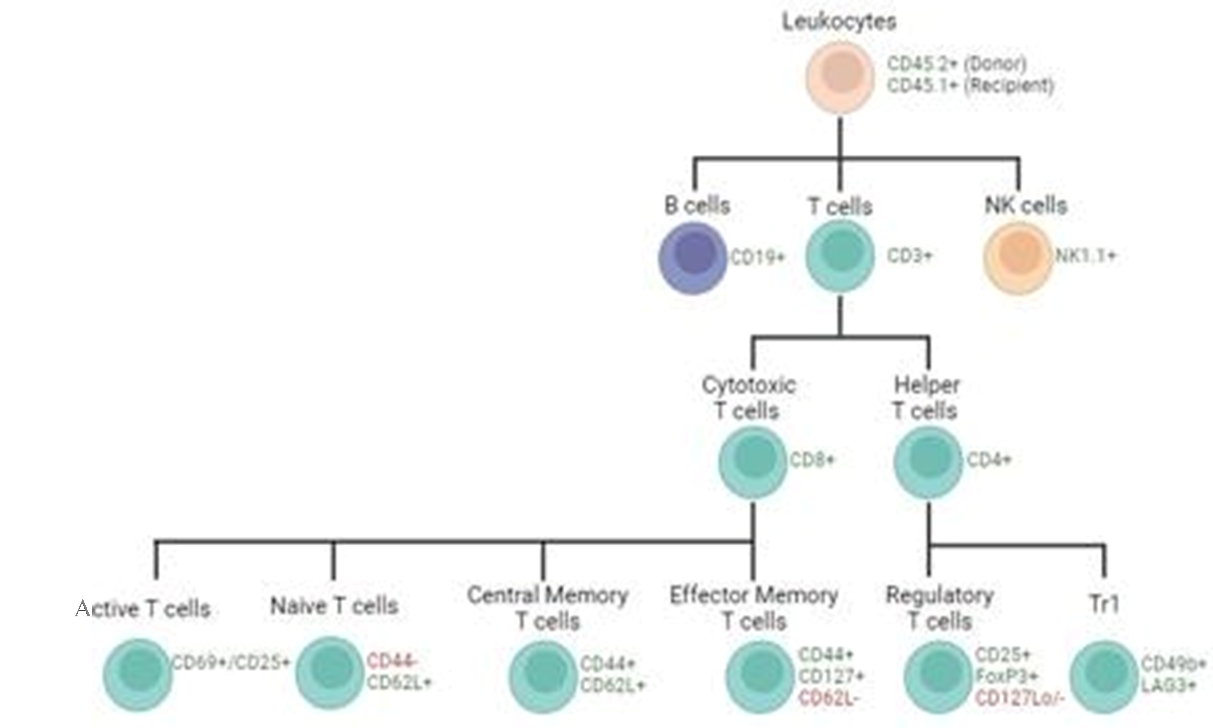
Supplementary Figures

##
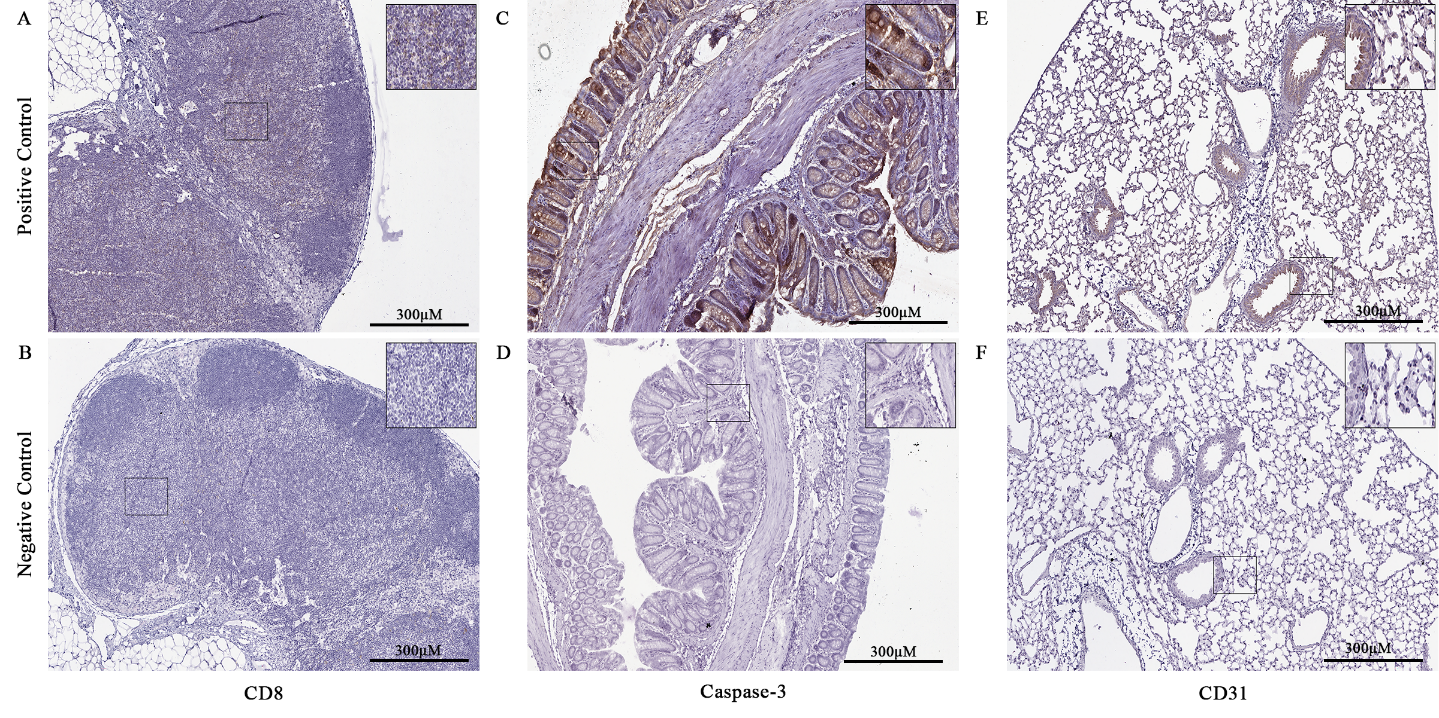
Supplementary Figure 1. Schematic of Immune Cell Populations and Markers Identified by Flow Cytometry Panel. The 16-colour flow cytometry panel will identify donor (CD45.2) and Recipient (CD45.1) cells, as well as respective NK cell, B cell, and various T cell subpopulations.

**Supplementary Figure 2.** Positive and Negative Controls for Immunohistochemistry. CD8 positive control (A) and negative control (B) using native mouse lymph node. Caspase-3 positive control (C) and negative control (D) using native mouse colon. CD31 positive control (E) and negative control (F) using native mouse lung. Scale bar: 300 µm. n = 2.

**
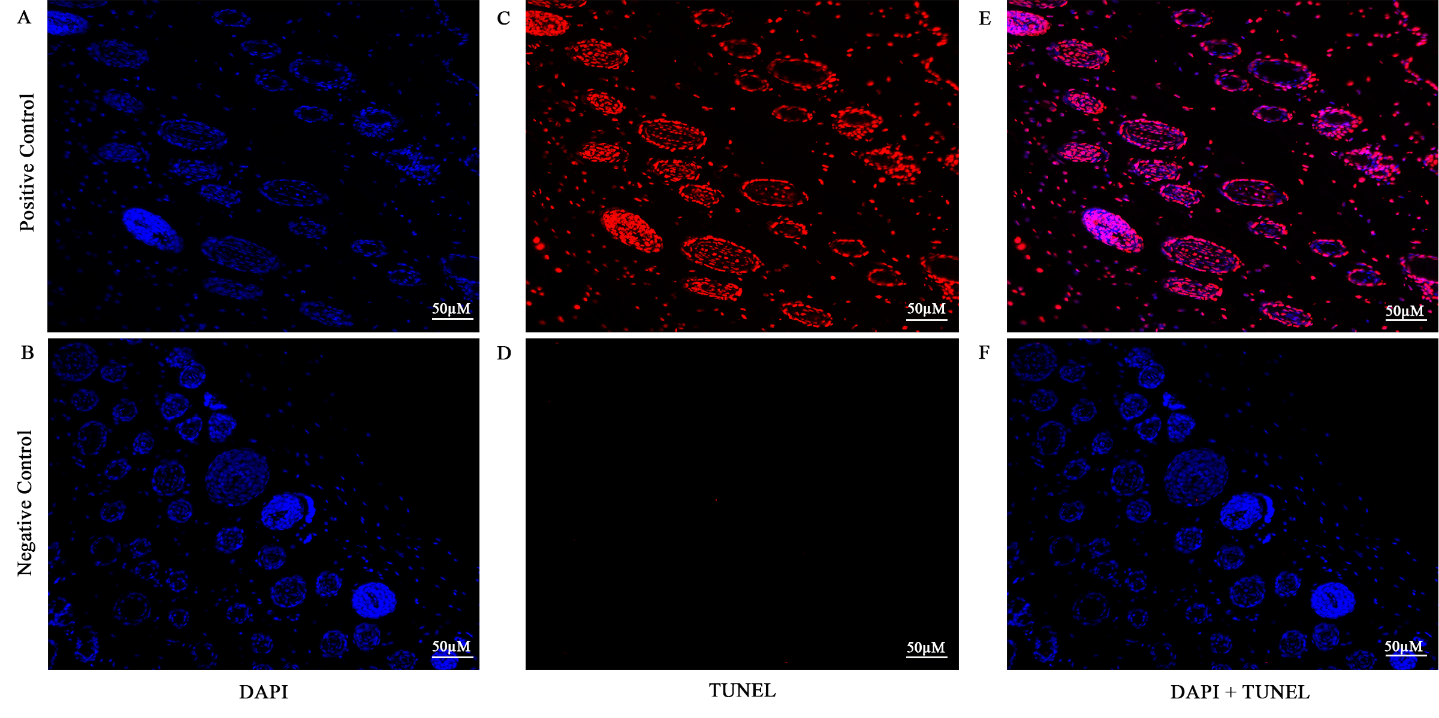
Supplementary Figure 3.** Positive and Negative Controls for TUNEL Staining in the Epidermis. Positive control was fixed with 1 mg/mL DNase I followed by staining with DAPI only **(A)**, TUNEL only **(C)**, and DAPI + TUNEL **(E)**. Negative control was treated with label solution without terminal transferase followed by staining with DAPI only **(B)**, TUNEL only **(D)**, and DAPI + TUNEL **(F)**. Scale bar: 50 µm. n = 3
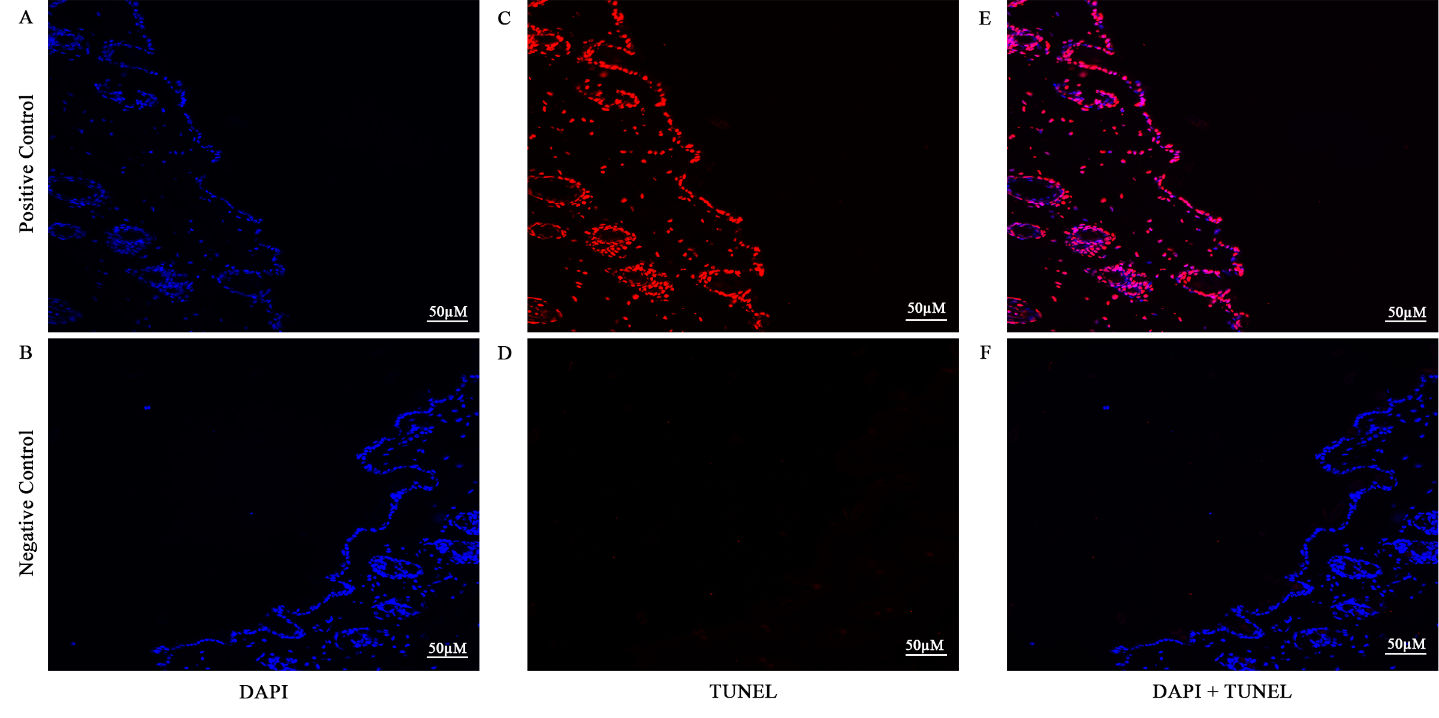
.

**Supplementary Figure 4.** Positive and Negative Controls for TUNEL Staining in the Dermis. Positive control was fixed with 1 mg/mL DNase I followed by staining with DAPI only **(A)**, TUNEL only **(C)**, and DAPI + TUNEL **(E)**. Negative control was treated with label solution without terminal transferase followed by staining with DAPI only **(B)**, TUNEL only **(D)**, and DAPI + TUNEL **(F)**. Scale bar: 50 µm. n = 3.


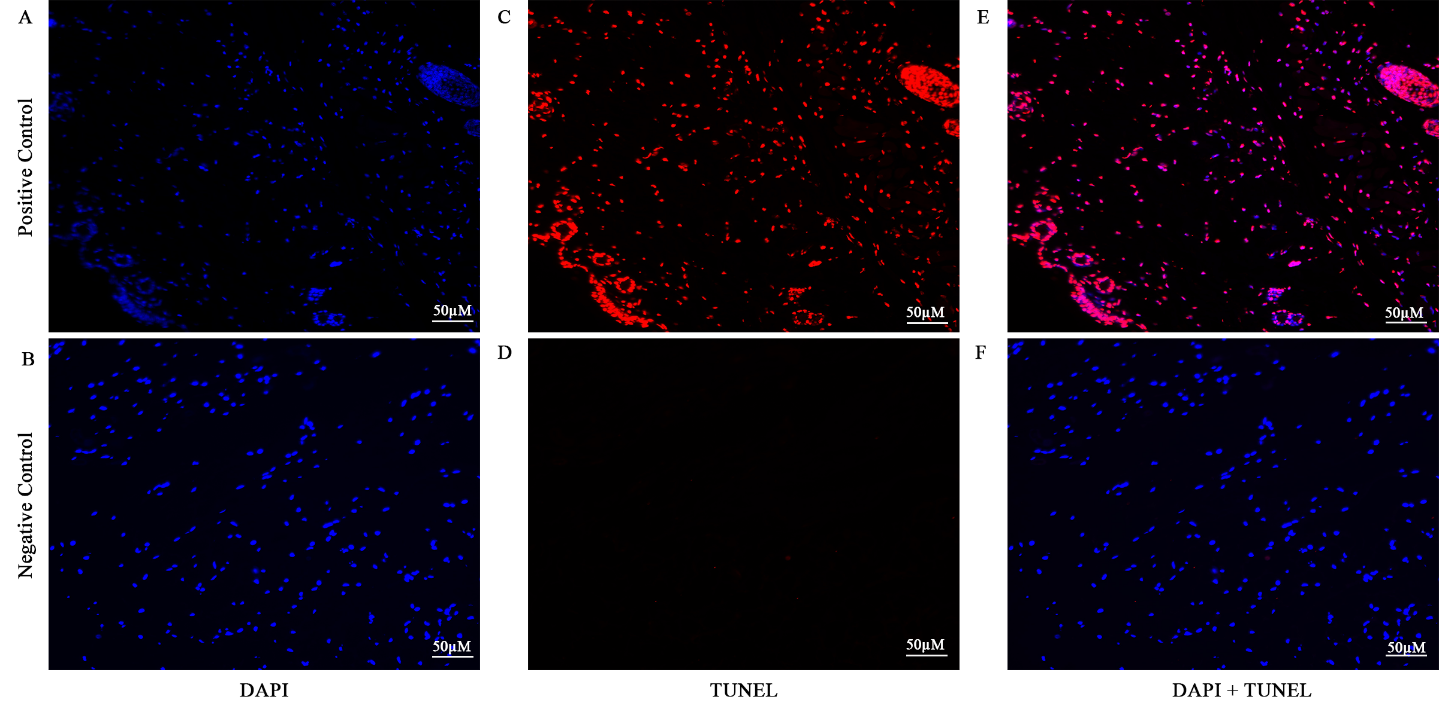

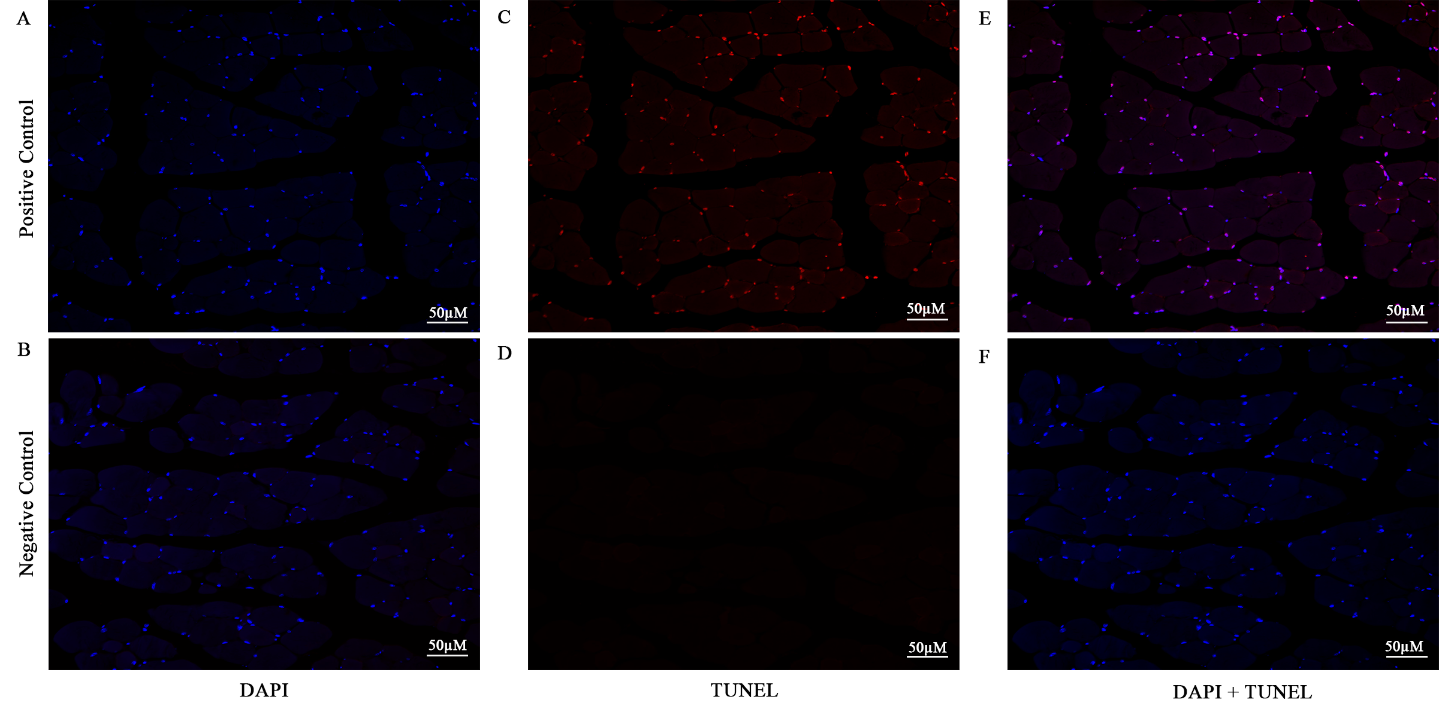
**Supplementary Figure 5.** Positive and Negative Controls for TUNEL Staining in the Deep-Dermis. Positive control was fixed with 1 mg/mL DNase I followed by staining with DAPI only **(A)**, TUNEL only **(C)**, and DAPI + TUNEL **(E)**. Negative control was treated with label solution without terminal transferase followed by staining with DAPI only **(B)**, TUNEL only **(D)**, and DAPI + TUNEL **(F)**. Scale bar: 50 µm. n = 3.

**Supplementary Figure 6.** Positive and Negative Controls for TUNEL Staining in the Muscle. Positive control was fixed with 1 mg/mL DNase I followed by staining with DAPI only **(A)**, TUNEL only **(C)**, and DAPI + TUNEL **(E)**. Negative control was treated with label solution without terminal transferase followed by staining with DAPI only **(B)**, TUNEL only **(D)**, and DAPI + TUNEL **(F)**. Scale bar: 50 µm. n = 3.


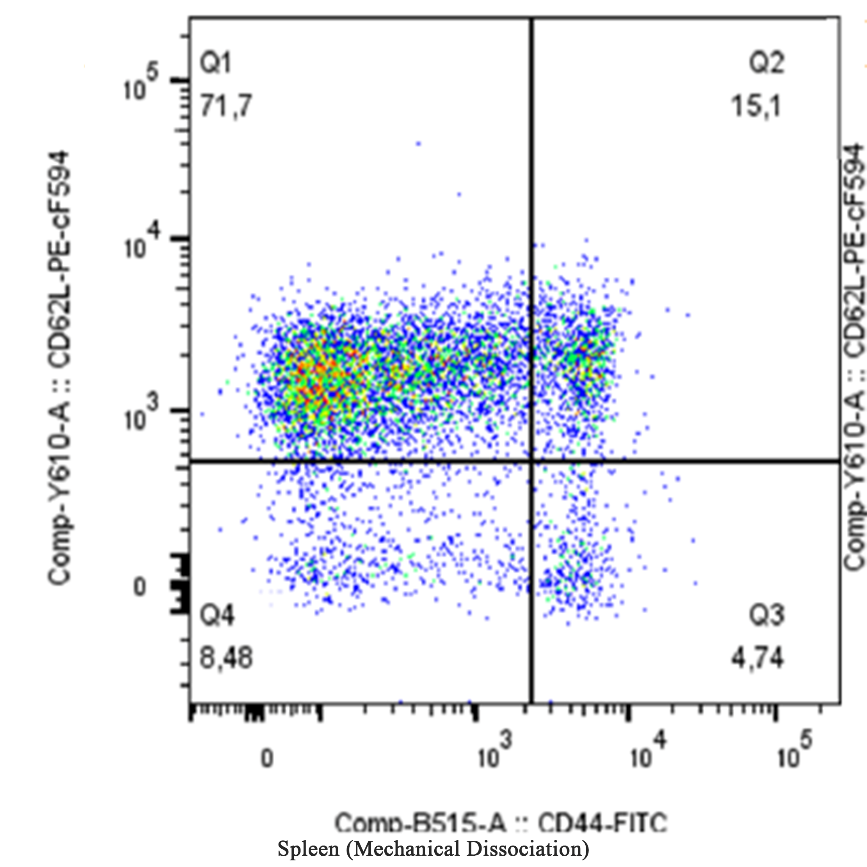


**Supplementary Figure 7.** Preservation of CD62L Marker via Skin Dissociation. Presence of CD62L in spleen cells dissociated using only mechanical dissociation. A total of 86.8% of cells are CD62L+. Cells are measured as a percentage of CD8+ cells. Fresh tissue samples were processed and fluorescently labelled prior to detection by flow cytometry using BD FACSymphony A3 Cell Analyzer. All data was analyzed using FlowJo (v10, FlowJo LLC).
